# Condition optimization of eco-friendly RP-HPLC and MCR methods via Box–Behnken design and six sigma approach for detecting antibiotic residues

**DOI:** 10.1038/s41598-023-40010-1

**Published:** 2023-09-21

**Authors:** Tahani Y. A. Alanazi, Rami Adel Pashameah, Ammena Y. Binsaleh, Mahmoud A. Mohamed, Hoda A. Ahmed, Hossam F. Nassar

**Affiliations:** 1 Chemistry Department, Faculty of Science, University of Ha’il, P.O. Box 2440, 81451 Ha’il, Saudi Arabia; 2 Department of Chemistry, Faculty of Applied Science, Umm Al-Qura University, 24230 Makkah, Saudi Arabia; 3 Department of Pharmacy Practice, College of Pharmacy, Princess Nourah Bint Abdulrahman University, P.O. Box 84428, 11671 Riyadh, Saudi Arabia; 4 Hikma Pharmaceutical Company, Beni-Suef, Egypt; 5 Department of Chemistry, Faculty of Science, Cairo University, Cairo, 12613 Egypt; 6 Environmental science and industrial development department, Faculty of Post Graduate Studies for Advanced Sciences, Beni-Suef University, Beni‑Suef, Egypt

**Keywords:** Analytical chemistry, Environmental chemistry

## Abstract

A precise, Eco-friendly, and highly sensitive RP-HPLC method was employed using quality-by-design principles to concurrently identify cephalexin and cefixime residues in the manufacturing machines using a hypersil BDS C18 column (250 × 4.6 mm, 5 μm) at wavelength 254 nm. The Box–Behnken design was applied to obtain the best chromatographic conditions with the fewest possible trials. Three independent factors viz organic composition, flow rate, and pH were used to assess their effects on the responses' resolution and retention time. Overlay plot and desirability functions were implemented to predict responses of the high resolution and relatively short retention time using a mobile phase composed of acidic water: acetonitrile (85:15, v/v) at pH 4.5 adjusted by phosphoric acid with a flow rate of 2.0 mL/min. The spectral overlapping of the drugs was successfully resolved by the mean centering ratio (MCR) spectra approach at 261 nm and 298 nm for cephalexin and cefixime, respectively. Good linearity results were obtained for the suggested HPLC and MCR methods over the concentration range of (0.05–10 ppm) and (5–30 ppm) with a detection limit of 0.003, 0.004, 0.26, and 0.23 ppm, and quantitation limits of 0.008, 0.013, 0.79, and 0.68 ppm for cephalexin and cefixime, respectively, with a correlation coefficient of ≥ 0.9998 and good swab recovery results of 99–99.5%. A process capability index was accomplished for chemical and micro results, illustrating that both are extremely capable. The suggested method was effectively validated using ICH recommendations.

A positive trend is toward recognizing the importance of environmentally sustainable practices in quantitative analysis. This is essential for the safety and well-being of analysts and for reducing the ecological impact of analytical processes. When developing new techniques, it is crucial to consider the method's sustainability, including the effects of solvents and waste production. The pharmaceutical industry has made impressive progress in promoting sustainability and environmental responsibility, which should encourage further efforts toward achieving even more tremendous strides in this area^1^. As we strive to improve our analytical methodologies' effectiveness, it is also essential to consider their potential environmental impacts. Fortunately, we can access various tools, including Analytical Method Volume Intensity (AMVI). Additionally, there are specialized tools such as Analytical Greenness (AGREE), Analytical Greenness for Sample Preparation (AGREEprep), Green Analytical Procedure Index (GAPI), Complementary Green Analytical Procedure Index (ComplexGAPI), and Analytical Eco-Scale (ESA), which have been designed to promote the adoption of sustainable analytical techniques. By incorporating these resources into our research, we can make meaningful contributions to scientific progress while fulfilling our responsibility to protect the environment^2^.

The potential risk of contamination of pharmaceutical drugs by various substances like microbe-related contaminants, residues from previous products, detergent residues, lubricants, auxiliary materials, such as disinfectants, and residues from decomposition. Effective cleaning practices are crucial for avoiding contamination and cross-contamination^3^. The goal of integrating the cleaning validation method is to provide proof that the cleaning process used for cleaning the solid suspension, capsule, and tablet lines is quite capable of removing traces of products residues that carried over from the prior product (worst-case product), microbiological contamination, detergent residues, and the cleaning method are efficient and repeatable^4^. The worst-case product should be chosen for cleaning validation if different Active pharmaceutical ingredients (APIs) were manufactured in the same machine that was cleaned using the same procedures. This selection relies on the rating criteria such as solubility, batch size, the difficulty of cleaning, maximum daily dose, and toxicity^5,6^. Pharmaceutical industries must prove, during validation, that the cleaning method used for a particular piece of equipment prevents potential carryover to a reasonable degree. The defined limitations were studied using sound scientific reasoning^7^. Cephalexin monohydrate (CPH) is 5-Thia-1-azabicyclo [4.2.0] oct-2-ene-2-carboxylic acid, 7-[(aminophenylacetyl)amino]-3-methyl-8-oxo-, monohydrate, [6*R*- [6α,7β (R*)]]-;(6*R*,7*R*)-7-[(*R*)-2 Amino-2-phenylacetamido]-3-methyl-8-oxo-5-thia-1-azabicyclo [4.2.0] oct-2-ene-2-carboxylic acid monohydrate. It is an antibiotic from the first generation of the cephalosporin group used to treat ear and respiratory infections. Its appearance is characterized as a powder that ranges in color from white to off-white and is crystalline. Extremely insoluble in alcohol, chloroform, and ether, only slightly soluble in water. As shown in Fig. S1a, its chemical formula is C_16_H_17_N_3_O_4_S · H_2_O, and its molecular weight is 365.40^8^. Cefixime trihydrate (CFX) is 5-Thia-1-azabicyclo[4.2.0]oct-2-ene-2-carboxylic acid, 7-[[(2-amino-4-thiazolyl)[(carboxymethoxy)imino]acetyl]amino]-3-ethenyl-8-oxo-, trihydrate, [6*R*-[6α,7β(*Z*)]]-;(6*R*,7*R*)-7-[2-(2-Amino-4-thiazolyl)glyoxylamido]-8-oxo-3-vinyl-5-thia-1-azabicyclo[4.2.0]oct-2-ene-2-carboxylic acid, 7^2^-(*Z*)-[*O*-(carboxymethyl)oxime]trihydrate. It is an antibiotic from the third generation of the cephalosporin family used to treat many bacterial infections. Its appearance is described as a powder ranging from white to pale yellow. Very insoluble in ether, ethyl acetate, hexane, and water. It is soluble in methanol and propylene glycol; it is only moderately soluble in alcohol, acetone, and glycerin; and it is only very partially soluble in 70% sorbitol and octanol. Following the data presented in Fig. S1b, it has the formula C_16_H_15_N_5_O_7_S_2_3H_2_O and a molecular weight of 507.50^8^.

Pharmaceutical companies are particularly interested in the benefits of using quality by design (QbD) for products and processes. The pharmaceutical industry is adopting QbD approaches to support strategies for constant progress and to increase the resilience of production processes, which support or improve product quality and manufacturing productivity. The concepts of QbD can be applied during method development and validation to confirm that the analytical method is established to achieve the desired quality standards^9^. Recently, the six sigma methodology was employed in most pharmaceutical companies to improve the process, reduce waste time, and detect errors before occurring to avoid reworking. The high value of the process capability index (Cpk) indicates that the process is capable. Furthermore, no additional work is needed^10^.

The literature review indicated that the analysis of CPH and CFX was officially described in the United States Pharmacopeia (USP) and British Pharmacopeia (BP) monograph using the HPLC method^8,11^. The quantification of CPH and CFX individually or in laboratory mixtures with other drugs has been mentioned using various analytical techniques such as HPLC^12–28^, LC–MS^19–35^, and Spectrophotometric methods^36–48^.

The novelty of the current work is to introduce accurate, specific, and credible RP-HPLC and MCR methods for detecting pharmaceutical drug residues of CPH and CFX in manufacturing machines using the QbD approach and green analytical chemistry metrics such as AMVI, ESA, AGREE, AGREEprep, GAPI, and ComplexGAPI that are beneficial for preserving safety and health, reducing the waste time, saving the cost of solvents and effort of analysts. Furthermore, the process capability index in lean six sigma methodology was accomplished for chemical and micro results with results of more than 1.3 to prove that method is suitable. As far as we know, HPLC and MCR techniques for simultaneously identifying pharmaceutical drug residues of CPH and CFX in manufacturing equipment employing the QbD methodology and six green analytical chemistry metrics have yet to be published. So, the primary goal of the current study is to guarantee the efficacy of the cleaning procedure for removing drug residues, product degradation, and detergent and to ensure no risk linked with cross-contamination of the drugs under investigation by employing the HPLC and MCR methods for simultaneous detection of CPH and CFX residues in environmental of production machines. Our study uses the Box–Behnken design and several analytical tools, such as AMVI, ESA, AGREE, AGREEprep, GAPI, and ComplexGAPI, to determine how drug quantification methods affect the environment. We aim to offer valuable insights that can advance the development of sustainable and eco-friendly approaches to drug quantification. This study significantly contributes to the field by prioritizing environmentally responsible practices.

## Experimental

### Instrument

HPLC system LC-20A (Shimadzu, Japan), including UV detector, column oven temperature, pump, degasser, autosampler, and contact with Empower 3 software to integrate and report the acquired data.

UV-1800 was manufactured by (Shimadzu, Japan) with high accuracy and sensitivity to comply with CFR part 11 for data integrity and supported with UV probe software 2.70 for data analysis and processing.

A vortex mixer in the shaker (BIOTEC-FISCHER, Germany) was used for blending samples in a test tube.

pH meter (Mettler-Toledo, Columbia, USA) was applied to estimate the pH in the solution.

Fluid Bed Dryer CPMFBD-120GMP (Ganson Pharma Machinery, India) was used for drying materials.

Vibro Sifter VS36 was manufactured by (Mill Power, India).

High-speed mixer (kneader) and Tablet press machine (compression) were manufactured by (Sejong Pharmatech, Korea).

Quality by design was evaluated by Design-Expert software version 13.

Minitab 2018 software was used to apply six sigma and the process capability index.

### Materials and reagent

CPH (Lot. No # HPI-WS/CPH-USP-23.09) and CFX (Lot. No # HPI-WS/CFX-USP-23.09) working standards were supplied from Hikma pharmaceutical industry (Beni-Suef, Egypt) with a potency of 98.4% and 100.8% as an anhydrous base according to their standardization against official primary USP standard of CPH (CAS# 23325-78-2) and CFX (CAS# 125110-14-7), respectively. Orthophosphoric acid (OPA) analytical grade and acetonitrile HPLC grade were purchased from (Scharlau, Spain).

### Procedures

#### Preparation of solvent

Acetonitrile: Purified water adjusted at pH 4.5 with OPA (20:80).

#### Preparation of working standard solutions (Laboratory prepared mixture)

To a volumetric flask of 200 mL, weigh about 25 mg CPH and CFX working standard, add 70% of diluent and sonicate till it dissolves, then complete the volume with the diluent. Transfer 4 mL into a 200 mL volumetric flask and achieve the volume with a diluent. (2.5 ppm). Filter the solution using a filter with a pore size of 0.22 μm.

#### Test preparation

Using a Plastic swab (Sterile swab stick) wetted with the diluent, swab 100 cm^2^ (10 × 10) of the target location following the pattern in (Supplementary Fig.  S2 online) and transfer the swab into a test tube containing 2 mL diluent. Then, vortex and sonic for 40 min. Filter the solution using a filter with a pore size of 0.22 μm.

#### Chromatographic conditions

Thermo hypersil C18 -BDS column (250 × 4.6 mm, 5 μm) and a mobile phase consisting of purified water: acetonitrile (85:15, v/v) at pH 4.5 adjusted by 0.1% OPA at ambient temperature and wavelength 254 nm with flow rate 2.0 mL/min and injection volume 100 µL were created for the concurrent analysis of the investigated drugs.

#### Calibration curves construction

The final concentration range of (0.05–10 ppm) and (5–30 ppm) for CPH and CFX in the suggested HPLC and MCR methods was successfully achieved by sequence dilution from a standard stock solution in 10 mL volumetric flasks. The sample set included an injection of system setup from the standard, five injections of system suitability, two injections of standard, two injections from blank, one injection of each cleaning validation sample, and two injections of the standard check. Data analysis and processing were established using empower software.

#### Box–Behnken design for enhancing HPLC methodology

One of the collections of the complex design of experiments (DOE) approaches is called the Box–Behnken Design (BBD). BBD offers a distinctive benefit over conventional using quadratic terms in the polynomial equation and can aid in enhancing responses and a better understanding of the interaction between independent factors. BBD lowers the effort of analysts and analysis costs by reducing the number of experimental trials. Three independent factors of flow rate, organic composition ratio, and pH were chosen, and retention time and resolution were selected as dependent responses, as shown in Table 1.

**Table 1:** Factors and responses for Box–Behnken experimental design of the optimized method.

Std	Run	Factor 1	Factor 2	Factor 3	Response 1	Response 2	Response 3
		A: Flow rate	B: ACN	C: pH	Rs	Rt _CPH_	Rt _CFX_
12	1	1.5	15	6	3.8	6.423	7.784
13	2	1.5	10	4.5	4	6.913	8.224
10	3	1.5	15	3	3.8	6.627	7.985
3	4	1	15	4.5	4.4	8.565	9.658
6	5	2	10	3	3	4.892	5.974
5	6	1	10	3	3.9	8.968	9.992
1	7	1	5	4.5	3.7	9.546	10.617
7	8	1	10	6	4.1	7.784	8.623
16	9	1.5	10	4.5	4	6.913	8.224
8	10	2	10	6	3	5.996	6.896
14	11	1.5	10	4.5	4	6.913	8.224
2	12	2	5	4.5	2.8	8.143	9.325
9	13	1.5	5	3	2.9	6.933	8.541
4	14	2	15	4.5	4.4	4.514	5.554
17	15	1.5	10	4.5	4	6.913	8.224
15	16	1.5	10	4.5	4	6.913	8.224
11	17	1.5	5	6	3.3	6.842	8.102

#### Selection of worst-case product

Worst-case product selection authenticates on several factors, including how difficult it is to clean, how soluble medications are in the water, the lowest effective dose in a single dosage form, and the toxicity of the materials. Based on the scoring for the above items, Suprax suspension, Keflex film-coated tablets, and Suprax capsules have been chosen as the worst-case products for their respective product lines.

#### Dirty and cleaning hold time

Dirty holding time (DHT) was established for one day or more before the start of cleaning to ensure that the cleaning could be successfully carried out after the specified time when the product remained on the surface of the equipment without further processing. Cleaning holding time (CHT) was estimated to be five days before the machine was prepared to receive the product.

#### Sampling procedure

The machine will be dried from any residual rinse water after the cleaning operation is complete before cleaning validation samples are taken. The apparatus and the area must be visually inspected to ensure no residue. The analytical method for the product will indicate the necessary sampling approach for API detection, and the geometry of the equipment might prefer one sampling technique over the other (flat versus convoluted surfaces, accessibility, surface area size). Priority was given to sample locations that are the most difficult to clean, such as windows, punches, corners, coves, portions with absorbent or porous surfaces, and parts with sustained product interaction. The chemical (active and detergent) samples were taken before the microbial samples. Swab sampling is generally preferred because it has better consistency and repeatability than rinse sampling. The action allows to physically remove of residues in addition to the dissolution that the swab diluent provides. Swab sampling entails applying an appropriate sample substance in a predetermined manner to a designated sampling surface (such as 25 cm^2^ or 100 cm^2^), such as in a zigzag pattern or ten strokes horizontally and vertically., as depicted in Fig. S2a,b. The sampling material is soaked with an appropriate solvent to enhance the quantitative take-up of residues. The quantification of residues involves the preparation of the sampling material according to specific processes, followed by analyzing the critical substance present in the eluate.

Sampling is typically conducted at critical locations within the production equipment. Rinse sampling entails purging the entirety of the equipment's product contact surface or specific components with an appropriate solvent, typically purified water. The critical substance in the rinsing fluid is analyzed to determine the number of residues. A designated quantity of purified water or solvent rinse is circulated through the sanitized surfaces of the specialized apparatus. Subsequently, the ultimate effluent is gathered and evaluated by the suggested High-Performance Liquid Chromatography (HPLC) technique. Rinse is limited to highly water-soluble substances using purified water as a solvent. API rinse sampling is used in case the hot spot and swabbed surfaces cannot be reached and swabbed.

#### Determination of maximum allowable carryover (MACO)

The worst-case limit should be calculated using the following since cleaning validation is based on worst-case scenarios; Product B selection will follow the minimum batch size/maximum daily dose ratio for all product lines. The worst case of the equipment train's full equipment size (of the same function) is used in conventional calculations. Use the strictest acceptance criteria.

#### Acceptance criteria based on health-based data ADE/PDE

Utilizing the available data on Acceptable Daily Exposure (ADE) or Permitted Daily Exposure (PDE) is recommended for the computation of the Maximum Allowable Carryover (MACO). The basic idea behind MACO is to determine how much of the prior product can be integrated into the new one while still meeting quality standards.

#### Acceptance criteria based on therapeutic daily dose (1/1000)

This computation can be employed when toxicity information is scarce and the Therapeutic Daily Dose (TDD) is ascertainable. This is utilized to transition the end product from API Process (A) to API Process (B). The typical maximum therapeutic daily dose of product B should be at most 1/1000 of the smallest adequate amount of product A.

#### Acceptance criteria based on LD50

The MACO can be calculated if just LD50 data is given (e.g., chemicals, intermediates, detergents).

#### General Limit as acceptance criteria (10 ppm)

A general limit may be appropriate if MACO calculations yield unacceptable carryover figures or no toxicological data for intermediates. Businesses may set a limit. The available limit sets a contaminated component's maximum concentration (MAXCONC) in a future batch.

#### Rinse test for detergent

The maximum allowable quantity of the used cleaning agent per day might be included in the most significant daily dose of the resulting product.

#### General Limit as acceptance criteria 1/10 of visual limit

The visual limit is commonly understood to be 400 µg/100 cm^2^. Tenth, the visible limit is equal to 40 µg/100 cm^2^.

#### Process capability index

The primary benefit of the process capability index is that it helps businesses gain insight into process behavior, leading to less waste, higher product quality and uniformity, and lower production expenses. In the face of significant variance, the process capability index (Cpk) reflects a process's proximity to its defined specification limit. The following formula can be used to determine how to raise the Cpk value, which indicates that the process requires tweaking^49^.

$${\text{Cpk }} = {\text{ Minimum }}\left( {\left( {{\text{X }} - {\text{LSL}}} \right)} \right)/{\text{3 or Minimum }}\left( {\left( {{\text{USL}} - {\text{X}}} \right)/{\text{3S}}} \right)$$

In this equation, LSL stands for the lower permitted limits, USL for the maximum allowed limit, and S for the process standard deviation.

Always, Cpk > Cp (Process Capability).

When the procedure is adequately aligned, Cpk = Cp.

A negative Cpk indicates the process’s mean is outside the allowed range.

With Cpk = 0, the process average is near the allowed range.

Cpk values below 1.0 indicate that the process falls short of the required standards.

A Cpk of 1 means that the process is within acceptable limits.

Most customers demand a Cpk of 1.33 or above (4 Sigma).

#### Mean centering of ratio spectra method

A sophisticated spectrophotometric technique was used to cope with the challenge of interfering between the spectra of CPH and CFX in a binary combination using only the mean centering ratio and no derivative steps^50^.

To illustrate the equation for mean centering, let us examine a vector in three dimensions (Z)^51^.

$$Z = \left[ {\begin{array}{*{20}c} 5 \\ 4 \\ 3 \\ \end{array} } \right]$$

Average Z-vector is

$$\overline{Z} = \left[ {\begin{array}{*{20}c} 4 \\ 4 \\ 4 \\ \end{array} } \right]$$

To express the mean centering of the vector Z, the expression

$${\text{MC}}\left( {\text{Z}} \right) = {\text{Z}} - {\overline{\text{Z}}} = \left[ {\begin{array}{*{20}c} 5 \\ 4 \\ 3 \\ \end{array} } \right] - \left[ {\begin{array}{*{20}c} 4 \\ 4 \\ 4 \\ \end{array} } \right] = \left[ {\begin{array}{*{20}c} { + 1} \\ 0 \\ { - 1} \\ \end{array} } \right]$$

It is easy to show that the mean center of a vector does not change when it is multiplied by a constant n and likewise when it is added to a vector v. The following expression can be used to get the sum absorbance of a binary mixture consisting of CPH and CFX when there is no interaction and Beer's low is followed:

1 $${\text{Abz}} = \alpha_{CPH} C_{CPH} + \alpha_{CFX} C_{CFX}$$

In this equation, $\mathrm{Abz}$ represents the absorbance vector of the mixture, ${C}_{CPH}$ and ${C}_{CFX}$ are the concentrations of the two drugs, and ${\alpha }_{CPH}$ and ${\alpha }_{CFX}$ are the molar absorptivities of these two drugs, respectively. By dividing the absorbance vector of the mixtures by ${\alpha }_{CPH}$, we obtain the first ratio spectrum (CFX).

2 $$CFX = \frac{{{\text{Abs}}\left( {{\text{Mix}}} \right)}}{{\alpha_{CPH} }} = \frac{{\alpha_{CFX} C_{CFX} }}{{\alpha_{CPH} { }}} + C_{CPH}$$

Instances where it is necessary to disregard the zero value so that division can proceed. When the mean is centered on a constant, the outcome is zero.

3 $${\text{MC}}\left( {C_{CFX} } \right) = {\text{MC}}\left( {C_{CPH} } \right) = { }0$$

Using the mean centering based on Eq. (2), therefore

4 $${\text{MC}}\left( {CFX} \right) = {\text{ MC}}\left[ {\frac{{\alpha_{CFX} C_{CFX} }}{{\alpha_{CPH} { }}}} \right]$$

The evaluation of the concentration of each drug is declared by Eq. (4), the statistical foundation of binary mixture analysis, with no overlap with the assessment of the concentration of the other drug. A standardization curve was generated for each sample or mixture by plotting MC(CFX) versus CFX concentration. The CPH standard curve was calculated similarly to the CFX ones.

## Results and discussion

### Preliminary study

The primary purpose of the proposed study is to develop a particular and reliable HPLC method for concurrently detecting binary mixtures of CPH and CFX residues in manufacturing machines with high precision and the shortest possible retention time. Several experiments were run to determine the optimal wavelength, column type, and mobile phase adjustment. The optimal wavelength, regarding high sensitivity and minimal noise, was found to be 254 nm after scanning at 200–400 nm for concentrations of 10 µg/mL of both drugs CPH and CFX, respectively, using solvent as blank, as shown in Fig. S3. There were several different columns tested, such as the Agilent ZORBAX Eclipse Plus C18 (250 mm × 4.6 mm, 5 µm), the GL Science Inertsil ODS-3 V (250 mm × 4.6 mm, 5 µm), and the Thermo hypersil C18 -BDS column (250 mm × 4.6 mm, 5 µm). Based on preliminary data, the Thermo hypersil C18 -BDS column (250 mm × 4.6 mm, 5 µm) most effectively separated the tested drugs with the smallest void volume. A mixture of acetonitrile and acidic water adjusted at pH 4.5 with 0.1% orthophosphoric acid worked brilliantly as a mobile phase for simultaneously estimating both pharmaceutical drugs. The retention time and resolution were quite sensitive to changes in acetonitrile concentration, pH, and flow rate. Because of their significant impacts on responses, flow rate, pH, and acetonitrile ratio in the mobile phase were highlighted as essential variables.

### Design of experiments (DoE)

The RSM was built utilizing a Design-of-Experiments (DoE) strategy called the BBD, which aids in determining the chromatographic parameters that enable optimal separation with minimal experimental trials and time investment while highlighting the significance of these factors and yielding second-order polynomial equations for the classifier of responses. Utilizing the BBD is a highly beneficial approach when optimizing chromatographic procedures for analysis. One of its primary advantages is the significant reduction in required experiments. However, it is essential to note that the BBD does not shield all possible combinations of variables at extreme levels, such as the highest or lowest. Therefore, evaluations conducted at those magnitudes could yield suboptimal outcomes. Although the BBD is an excellent tool for procedure optimization, it is not recommended for obtaining information about the response at the extreme values of independent variables^52^. All tests included five iterations of zero-level (pH 4.5, Flow rate 1.5, and Acetonitrile ratio 10%) factors estimation to ascertain the pure errors. The remaining 12 runs were randomized to minimize the influence of external variables that may lead to biased results. The linear regression analysis generated the following second-order polynomial equations showing the link between the responses and the predictor variable^53^.

5 $$\begin{aligned} {\text{RS}} & = + 4.00 - 0.3625{\text{ F }} + 0.4625{\text{ ACN}} + 0.0750{\text{ pH}} \\ & \quad + 0.2250{\text{ F}}*{\text{ACN}} - 0.0500{\text{ F}}*{\text{pH }} - 0.1000{\text{ ACN}}*{\text{pH}} \\ & \quad - 0.0625{\text{ F}^{2}} - 0.1125{\text{ACN}^{2}} \, - 0.4375{\text{ pH}^{2}} \\ \end{aligned}$$

6 $$\begin{aligned} {\text{Rt}}_{{{\text{CPH}}}} & = + 6.91 - 1.41{\text{ F}} - 0.6669{\text{ ACN }} - 0.0469{\text{ pH}} \\ & \quad - 0.6620{\text{ F}}*{\text{ACN}} + 0.5720{\text{ F}}*{\text{pH }} - 0.0282{\text{ ACN}}*{\text{pH }} \\ & \quad + 0.4914{\text{ F}^{2}} \, + 0.2876{\text{ ACN}^{2}} \, - 0.4944{\text{ pH}^{2}} \\ \end{aligned}$$

7 $$\begin{aligned} {\text{Rt}}_{{{\text{CFX}}}} = & + 8.22 - 1.39{\text{ F}} - 0.7005{\text{ ACN}} - 0.1359{\text{ pH}} \\ & \quad - 0.7030{\text{ F}}*{\text{ACN}} + 0.5728{\text{ F}}*{\text{pH}} + 0.0595{\text{ ACN}}*{\text{pH }} \\ & \quad + 0.1664{\text{ F}^{2}} \, + 0.3981{\text{ ACN}^{2}} \, - 0.5191{\text{ pH}^{2}} \\ \end{aligned}$$

where RS is the resolution response between CPH and CFX, Rt _CPH_ and Rt _CFX_ are the retention time responses for CPH and CFX, respectively. F (flow rate), ACN (acetonitrile ratio%), pH (pH of buffer), F*ACN, F*pH, and ACN*pH reflect the interaction of the variables, while F^2^, ACN^2^, and pH^2^ are the quadratic term.

### A statistical modeling approach

If the *P*-value for the model and its terms is less than 0.05, then they are statistically significant. The regression models' R-squared and adjusted R-squared values were within the acceptable ranges (R > 0.8), indicating a proper fit with a polynomial equation and facilitating the model’s predictive capability estimate. Appropriate data matching is characterized by high R-square and adjusted R-square values, whereas high forecasted R-squared values demonstrate the model's vital forecasting accuracy for future estimations^54,55^.

### Influences of variables

**Figure 1:**
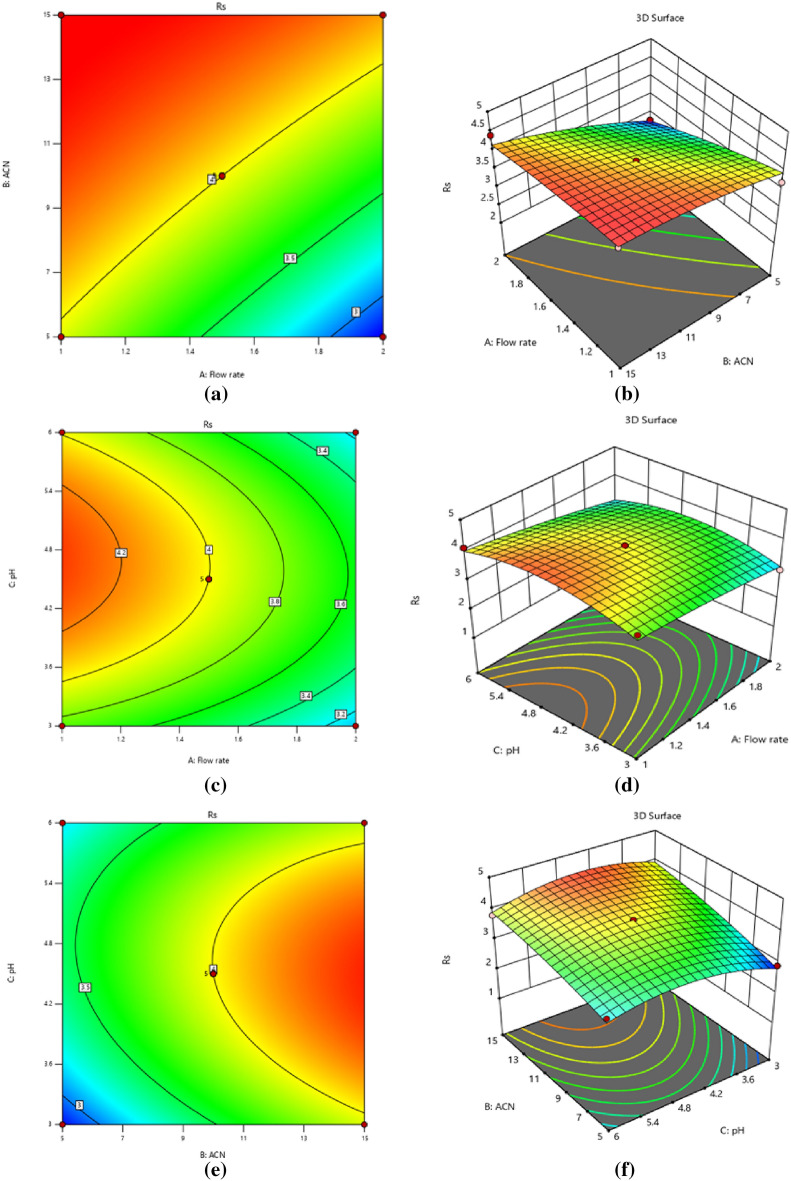
Contour and 3D-response surface plots (**a**, **b**) the effects of (% ACN) and flow rate, (**c**, **d**) pH and flow rate, (**e**, **f**) pH and (% ACN) on resolution, respectively.

**Figure 2:**
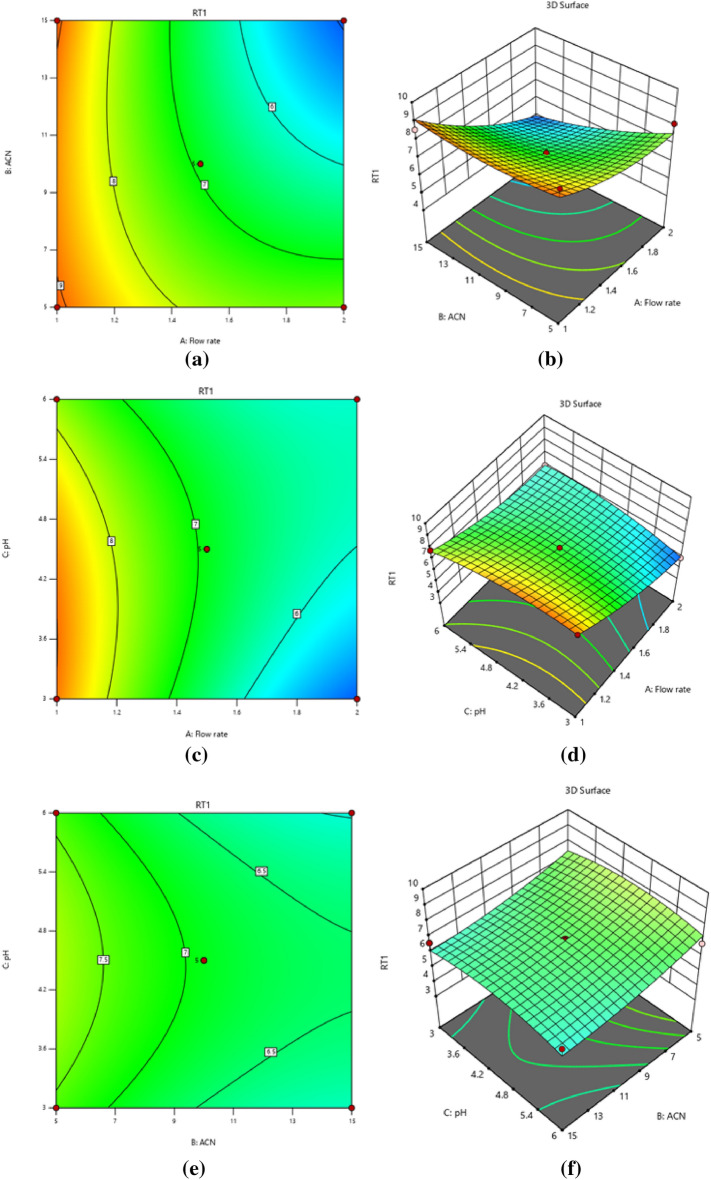

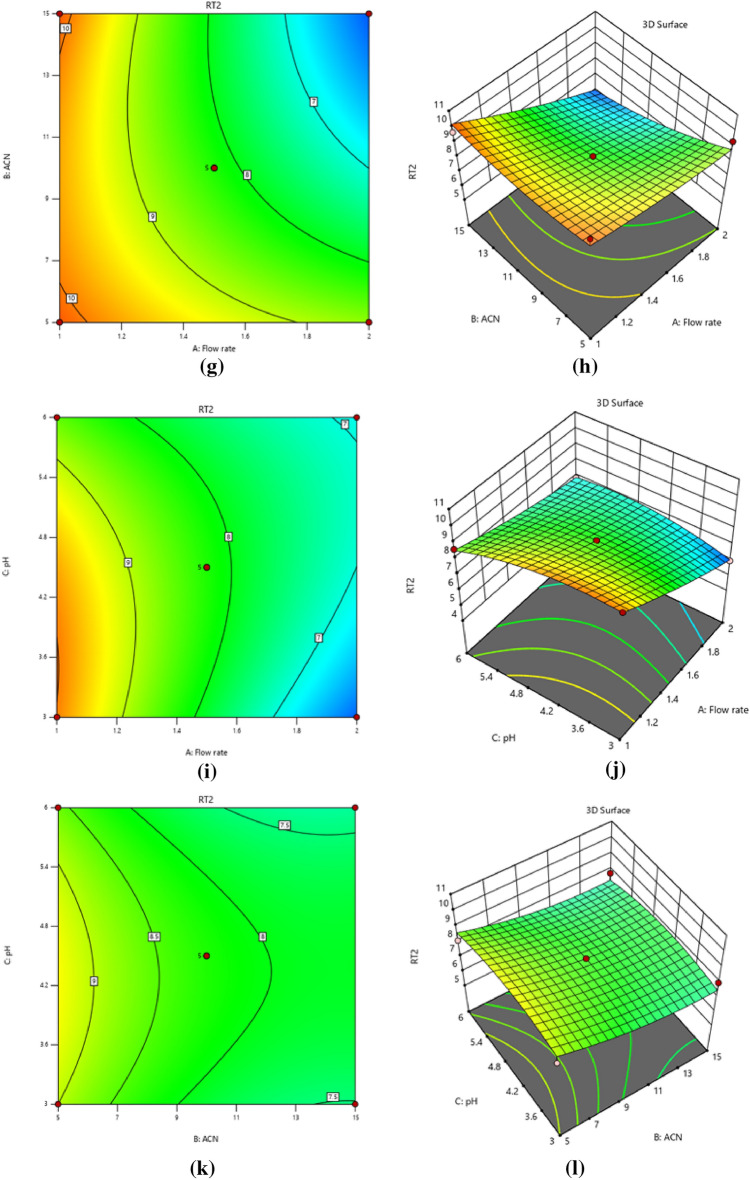
Contour and 3D-response surface plots (**a**, **b**) the effects of (% ACN) and flow rate, (**c**, **d**) pH and flow rate, (**e**, **f**) pH and (% ACN) on the retention time of CPH, respectively. The effects of flow rate and (% ACN) (**g**, **h**), (**i**, **j**) pH and flow rate, (**k**, **l**) pH and (% ACN) on the retention time of CFX.

Coefficients with positive signs in regression equations show that the resolution response is positively related to the independent variable; for example, the resolution response is positively associated with the ratio of acetonitrile to buffer pH and inversely associated with the flow rate, while the CPH and CFX Retention time responses are negatively associated with the predictor factors. Resolution response is positively affected by a linear buffer pH and acetonitrile ratio. However, it has a negative quadratic influence, meaning that the resolution response grows larger as pH rises to a critical point, after which further increases in pH reduce the response. The linear effect of variables on CPH and CFX retention time responses is negative. Still, its quadratic impact is positive, showing that the retention time response reduces as variables rise from low to high. The sign before the interacting terms, which is always positive, shows that the two variables respond similarly; in this case, decreasing the flow rate increases the resolution response. The amount of acetonitrile is also maintained to a minimum. In addition, the negative result shows that the two predictor variables act inversely, e.g., the response is reduced by raising the buffer's pH while maintaining a low flow rate. Three-dimensional surface plots and two-dimensional contour plots illustrate the graphical depiction of Eq. (1) in Fig. 1a,b, which displayed the effects of acetonitrile ratio and flow rate, Fig. 1c,d, the impact of pH and flow rate, and Fig. 1e,f, the results of pH and acetonitrile ratio on resolution, respectively. And Eqs. (2, 3) in Fig. 2a,b, which depicted the influences of acetonitrile ratio and flow rate, Fig. 2c,d, the effects of pH and flow rate, and Fig. 2e,f, the effects of pH and acetonitrile ratio on the retention time of CPH, respectively. The impact of flow rate and acetonitrile ratio in Fig. 2 g, h, pH and flow rate in Fig. 2i,j, pH and acetonitrile ratio in Fig. 2k,l on the retention time of CFX, which show the hypothetical interaction between the two predictor factors and the responses while holding the third factor unchanged. The parabolic curve of the contour plots represents the non-linear relationships between the factors and the outcomes. Tables 2 and 3 provide ANOVA results for responses based on resolution and retention time; a probability P-value of less than 0.05 indicates that the model and terms are significant. Resolution and retention time responses achieved R-squared and adjusted values of 0.94, 0.86, 0.93, and 0.84, respectively, while standard deviations were less than 0.53 and lack of fit values were 0.258 and 1.91, and 1.88. These results indicate that experimental responses were typically suitable.

**Table 2:** ANOVA data for resolution response.

Source	Sum of squares	df	Mean square	F-value	*P*-value	
Model	3.98	9	0.4422	12.02	0.0017	Significant
A-Flow rate	1.05	1	1.05	28.58	0.0011	
B-ACN	1.71	1	1.71	46.52	0.0002	
C-pH	0.0450	1	0.0450	1.22	0.3053	
AB	0.2025	1	0.2025	5.50	0.0514	
AC	0.0100	1	0.0100	0.2718	0.6182	
BC	0.0400	1	0.0400	1.09	0.3317	
A^2^	0.0164	1	0.0164	0.4471	0.5251	
B^2^	0.0533	1	0.0533	1.45	0.2679	
C^2^	0.8059	1	0.8059	21.91	0.0023	
Residual	0.2575	7	0.0368			
Lack of fit	0.2575	3	0.0858			
Pure error	0.0000	4	0.0000			
Cor total	4.24	16

**Table 3:** ANOVA data for a retention time of CPH and CFX responses.

Source	Sum of squares	df	Mean square	F-value	*P*-value	
*Rt of CPH*						
Model	24.95	9	2.77	10.14	0.0029	Significant
A-Flow rate	16.01	1	16.01	58.58	0.0001	
B-ACN	3.56	1	3.56	13.02	0.0087	
C-pH	0.0176	1	0.0176	0.0643	0.8071	
AB	1.75	1	1.75	6.41	0.0391	
AC	1.31	1	1.31	4.79	0.0649	
BC	0.0032	1	0.0032	0.0117	0.9170	
A^2^	1.02	1	1.02	3.72	0.0951	
B^2^	0.3483	1	0.3483	1.27	0.2962	
C^2^	1.03	1	1.03	3.76	0.0935	
Residual	1.91	7	0.2733			
Lack of fit	1.91	3	0.6378			
Pure error	0.0000	4	0.0000			
Cor total	26.86	16				
*Rt of CFX*						
Model	24.71	9	2.75	10.23	0.0029	Significant
A-flow rate	15.52	1	15.52	57.82	0.0001	
B-ACN	3.93	1	3.93	14.63	0.0065	
C-pH	0.1477	1	0.1477	0.5504	0.4823	
AB	1.98	1	1.98	7.37	0.0300	
AC	1.31	1	1.31	4.89	0.0627	
BC	0.0142	1	0.0142	0.0528	0.8249	
A^2^	0.1166	1	0.1166	0.4343	0.5310	
B^2^	0.6674	1	0.6674	2.49	0.1588	
C^2^	1.13	1	1.13	4.23	0.0788	
Residual	1.88	7	0.2684			
Lack of fit	1.88	3	0.6262			
Pure error	0.0000	4	0.0000			
Cor total	26.59	16				

### The function of composite desirableness

**Figure 3:**
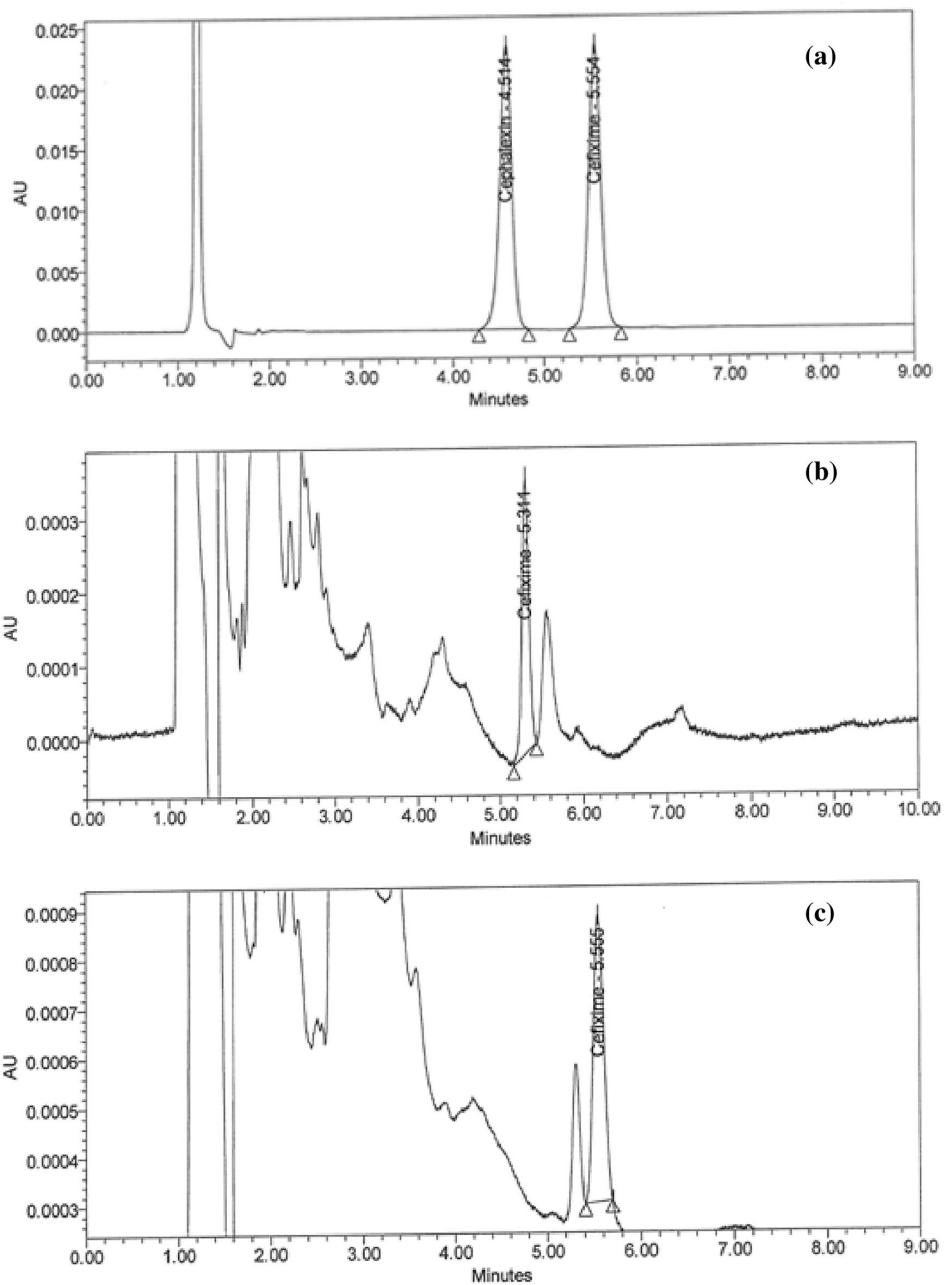

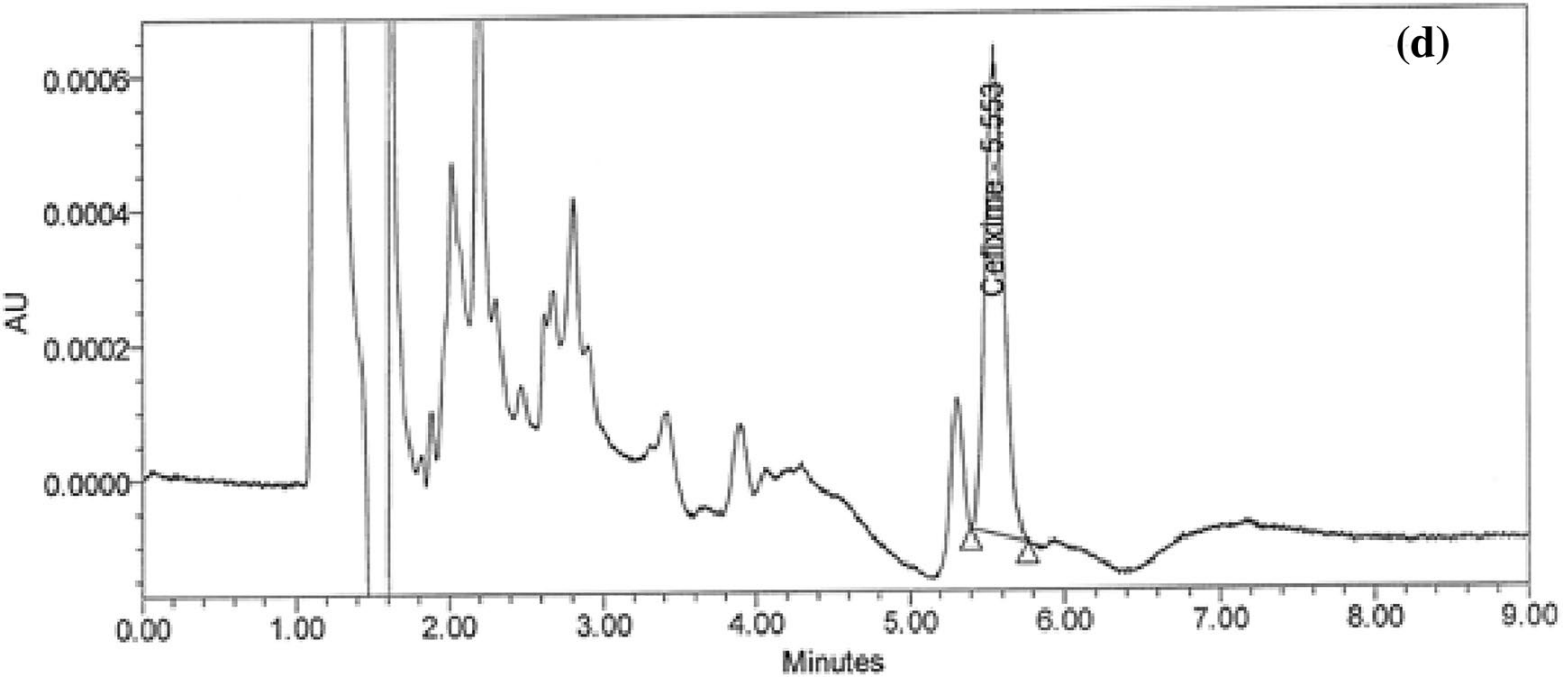
HPLC Chromatograms of (**a**) 2.5 ppm for the standard solution of a mixture of CPH and CFX and 0.23, 0.29, and 0.22 ppm of CFX residues in the (**b**) sieve, (**c**) pan, and (**d**) product discharge parts, respectively in the sifter machine.

In predicting the responses and achieving the optimal separation criteria by increasing desirability to acquire high resolution > 4.0 and retention time shorter than 6.0 min, the numerical optimization function was utilized, as shown in (Supplementary Fig. S4a–c online). A series of overlay plots highlighted the most influential factors in achieving the desired results (Supplementary Fig. S4d–f online). The hypothesized approach was verified in the lab by implementing various parameters. As shown in Fig. 3a,b–d), the best chromatographic system used to have a high resolution, an asymmetric peak, and a shorter retention time when using acidic water: acetonitrile (85:15, v/v) at pH 4.5 adjusted by the phosphoric acid at a flow rate of 2.0 mL/min.

### Mean centering of ratio spectra method

**Figure 4:**
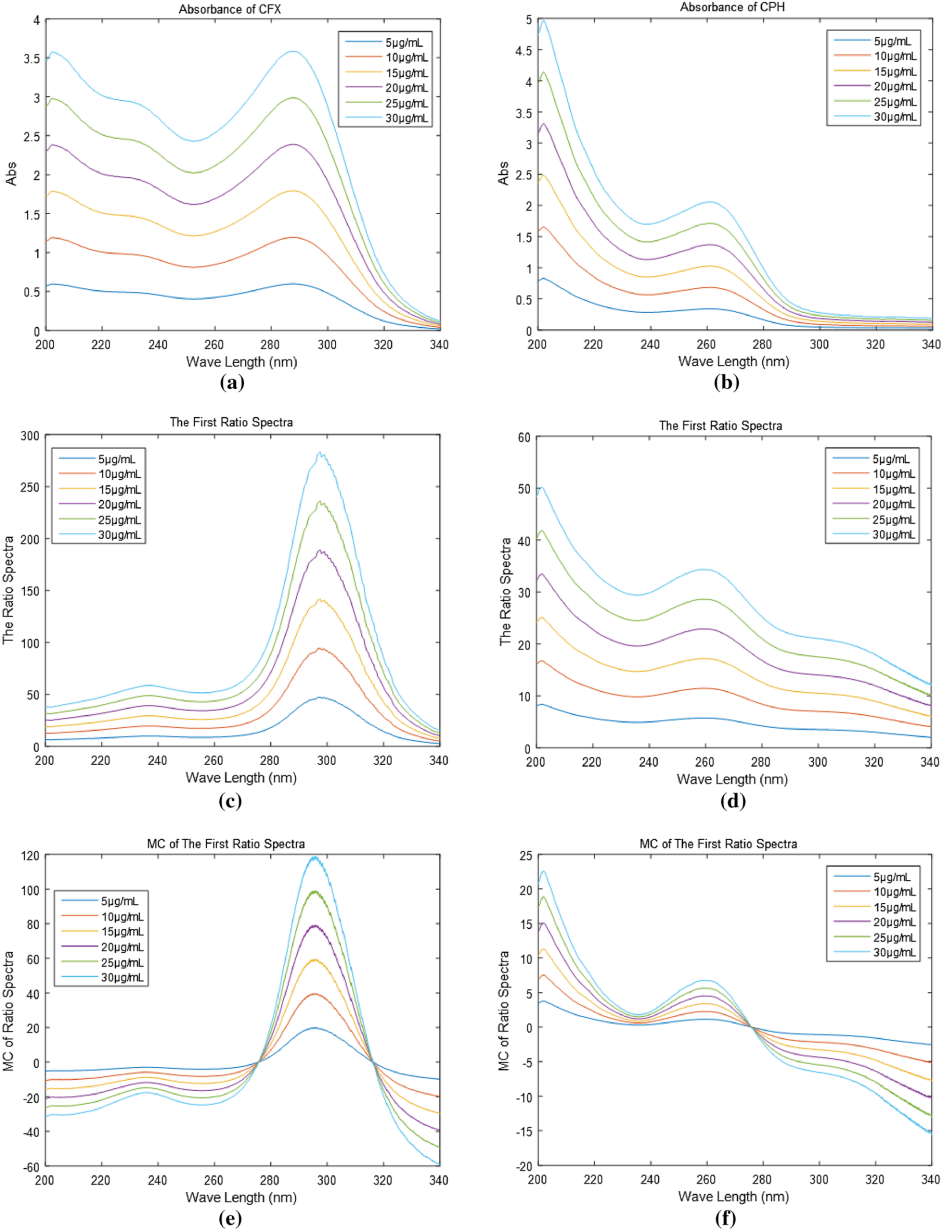
(**a**, **b**) (5–30 µg/mL) absorbance of CFX and CPH, (**c**, **d**) First ratio spectra of CFX and CPH, and (e, f) MCR spectra of CFX and CPH.

Obtaining the MC of ratio spectra is the foundation of the established MCRS technique. A CPH and CFX mixture is analyzed using the MCRS method; no pre-separation is required. The highly selective method was developed by testing a variety of divisor concentrations to find the optimal one. The optimal concentration is 10 µg/mL. The spectra of the drugs presented in Fig. 4a–d were generated in the wavelength range of 200–340 nm. The spectra that have been stored, which represent various drug concentrations, are then imported into the MATLAB software. Applying equations one through four, the concentrations of CPH and CFX in the actual binary mixture can be calculated. Based on the graphs of MC of first ratio spectra for the drugs that were generated, as shown in Fig. 4e,f, it has been determined that the appropriate wavelength for the determination of CPH and CFX, respectively, is 261 nm and 298 nm. Plotting the observed amplitudes against their respective concentrations yields the standard curves, and Table 4 shows the correlation coefficients.

**Table 4:** Descriptive statistics for the process capability six-pack of the chemical and micro results.

Parameters	Results	
Descriptive statistic	Chemical	Micro
Minimum	0.17	11
Maximum	0.31	21
Mean	0.238	14.91
Limit	NMT 5.0 ppm	NMT 50 ppm
Sum	20.00	1252
Count	84	84
Standard error	0.004	0.31
Median	0.235	14
Mode	0.23	12
Standard deviation	0.04	2.81
Variance	0.001	7.92
Kurtosis	− 1.06	− 0.85
Skewness	− 0.04	0.53
Range	0.14	10

### Handling of cleaning validation samples

**Figure 5:**
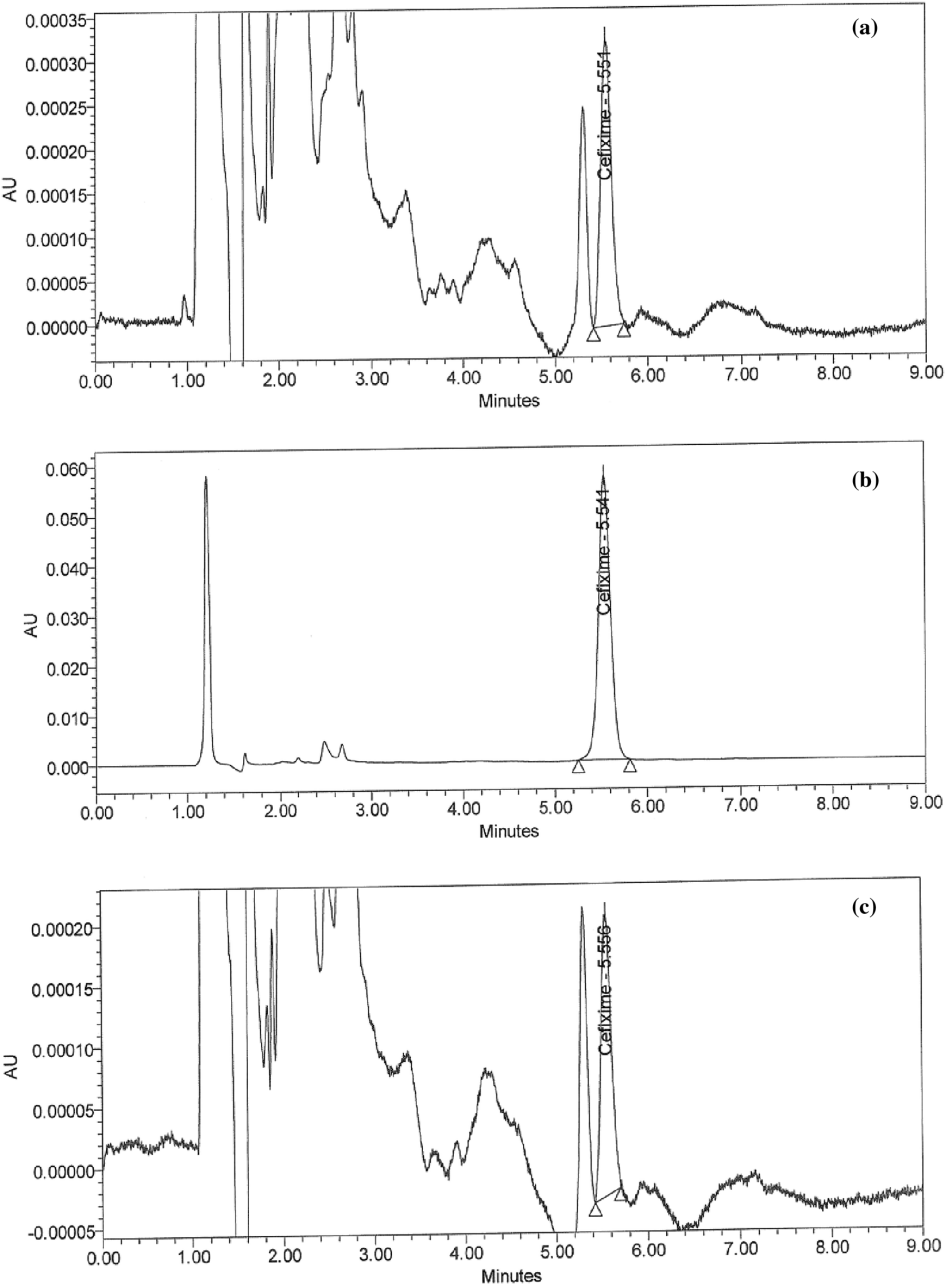

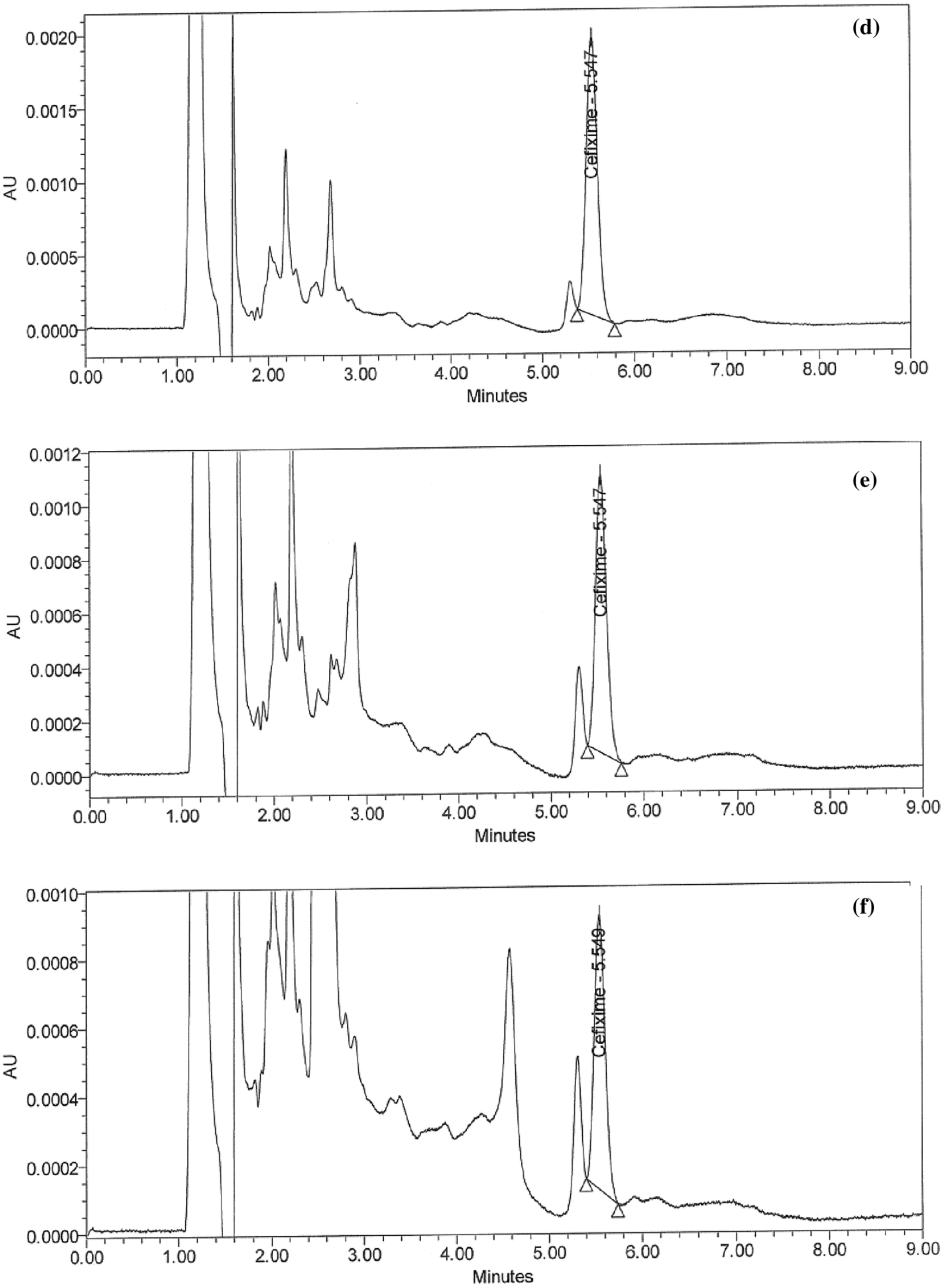

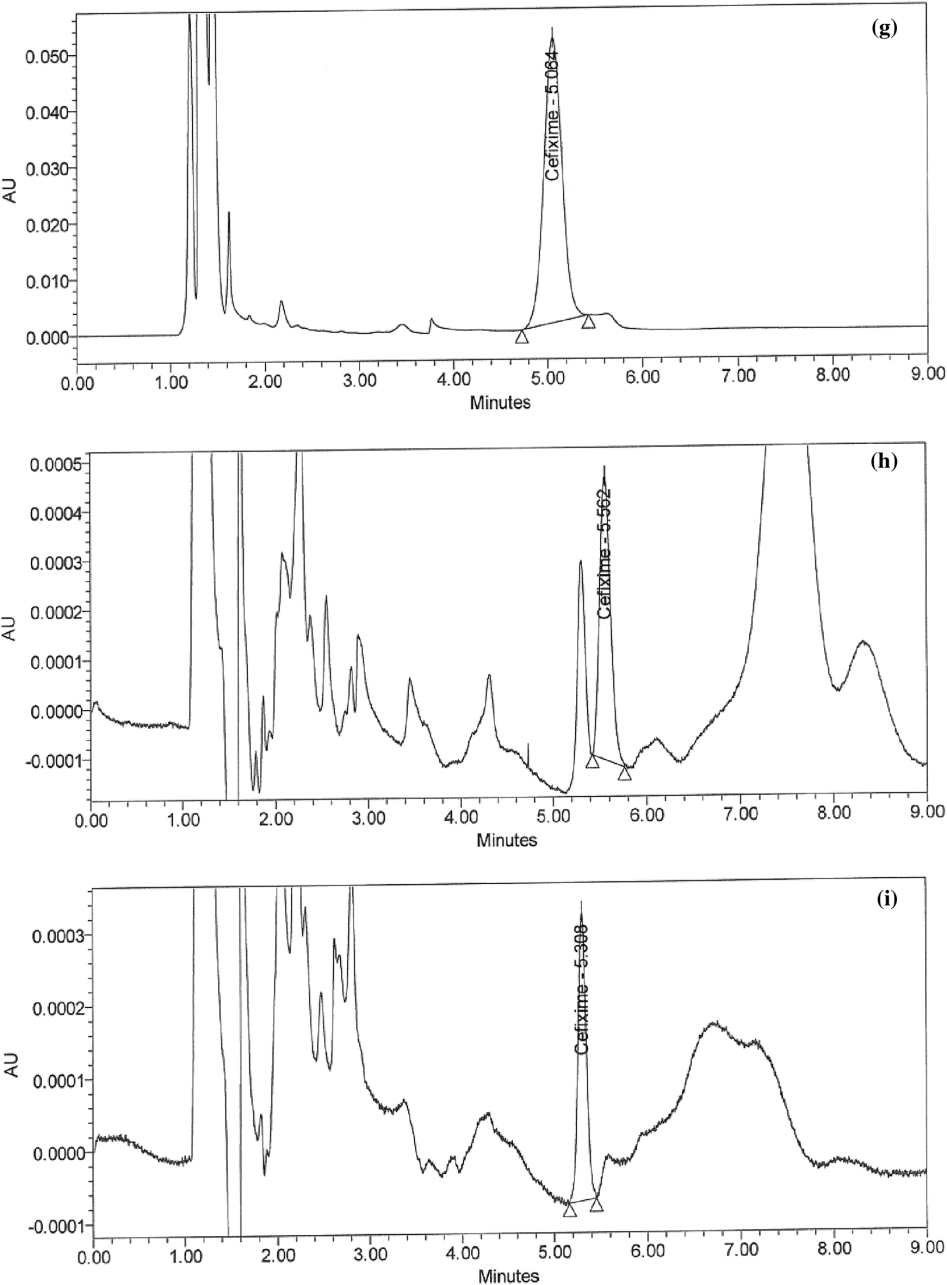

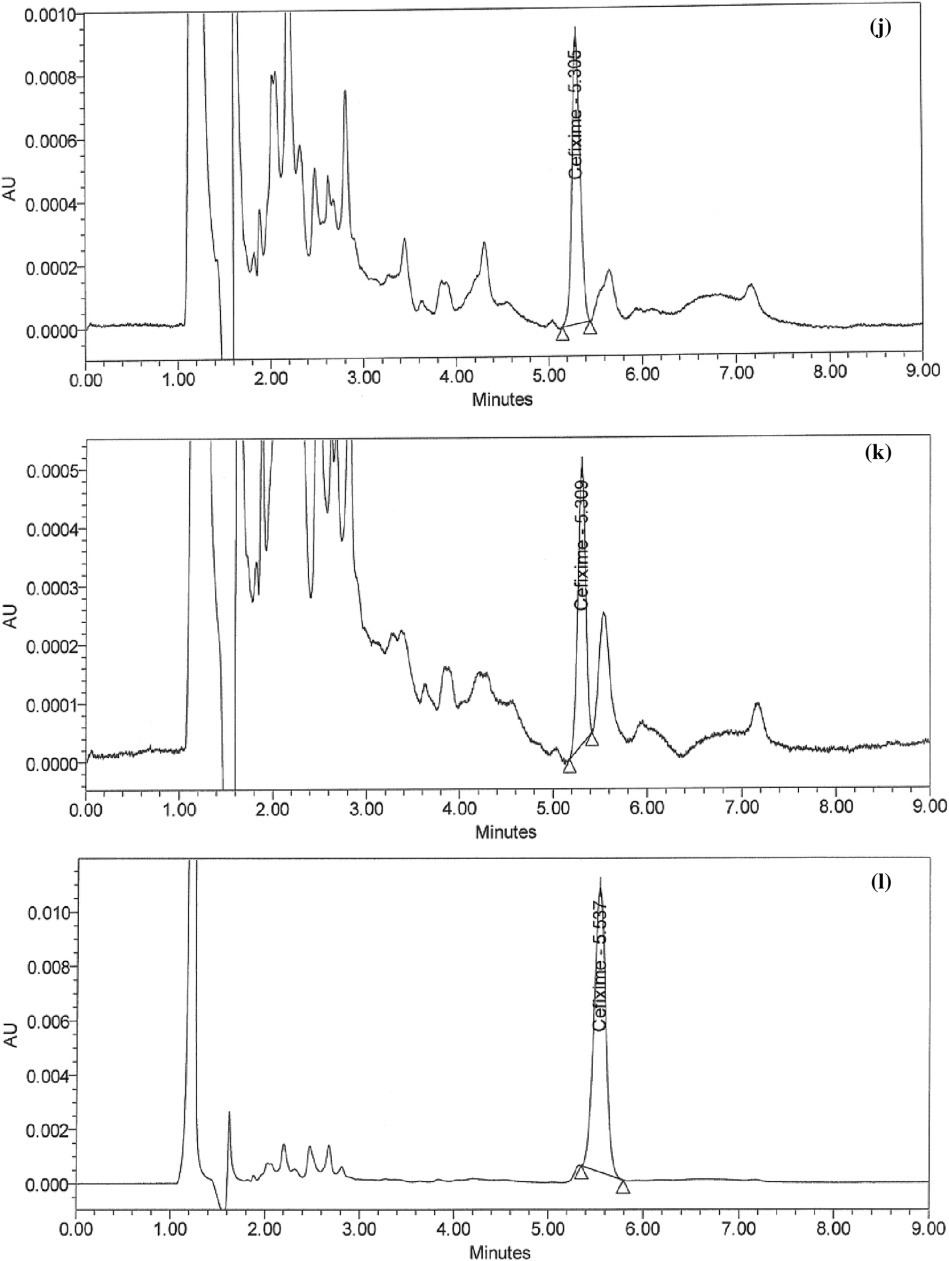
HPLC Chromatograms of CFX residues in (**a**–**l**) compression machine parts.

**Figure 6:**
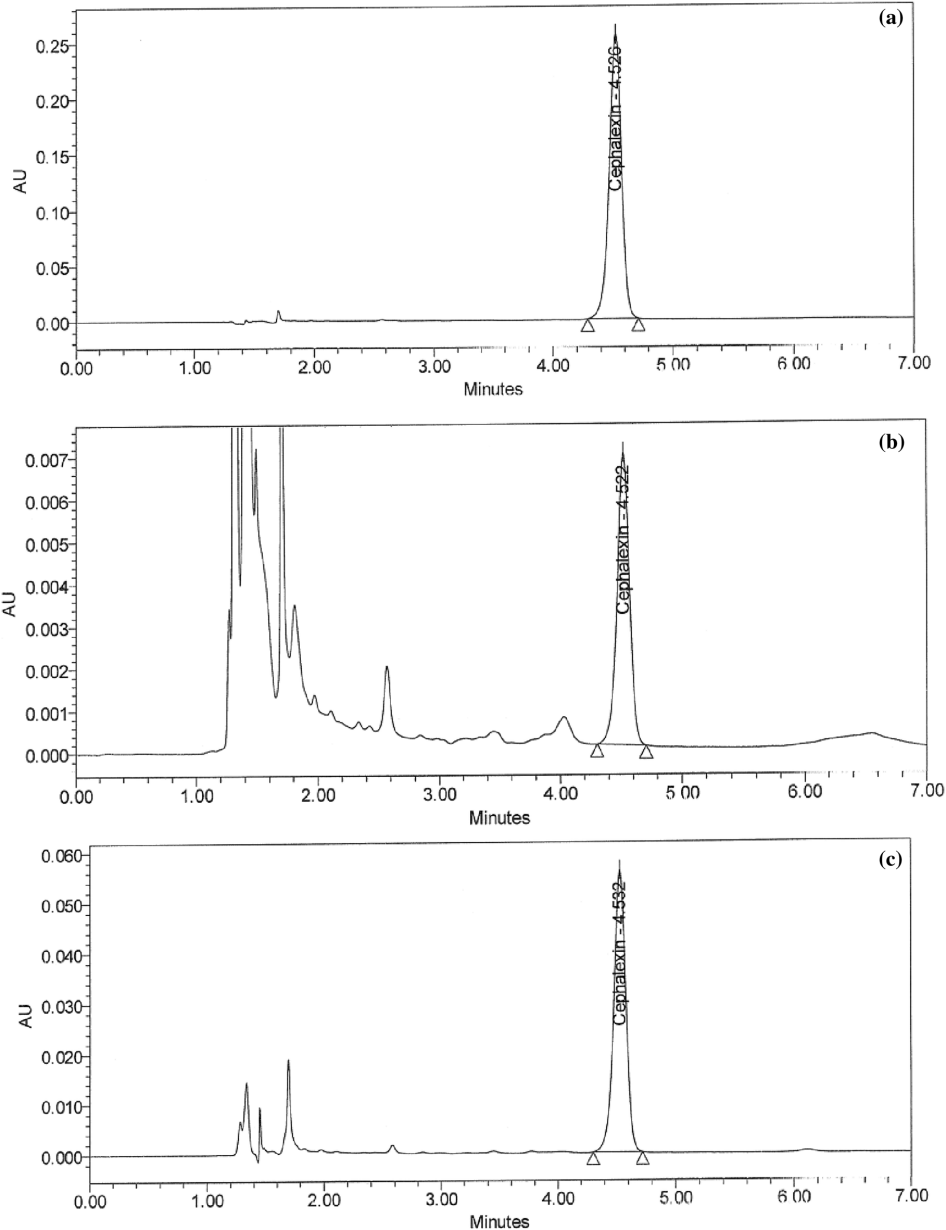

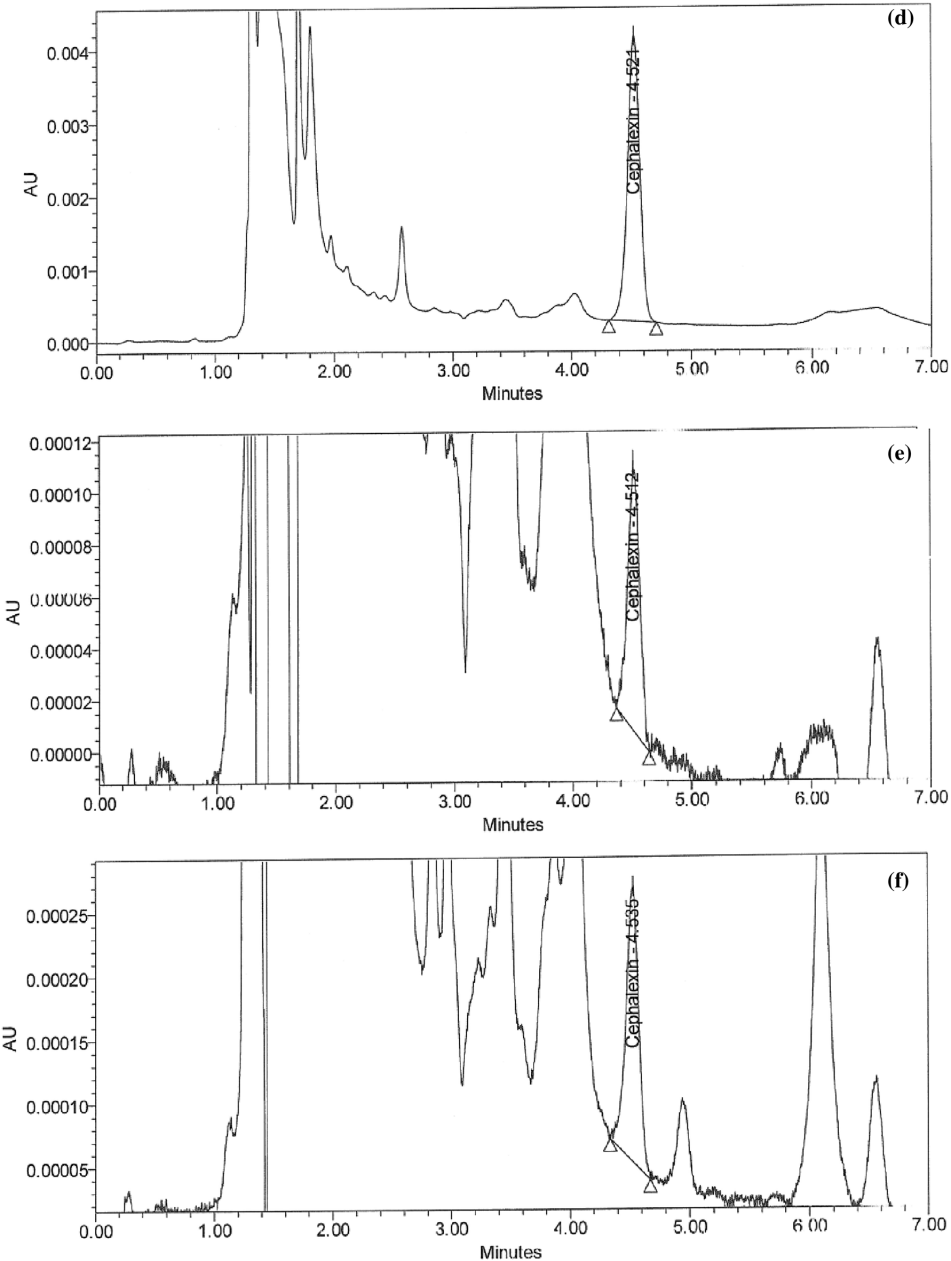

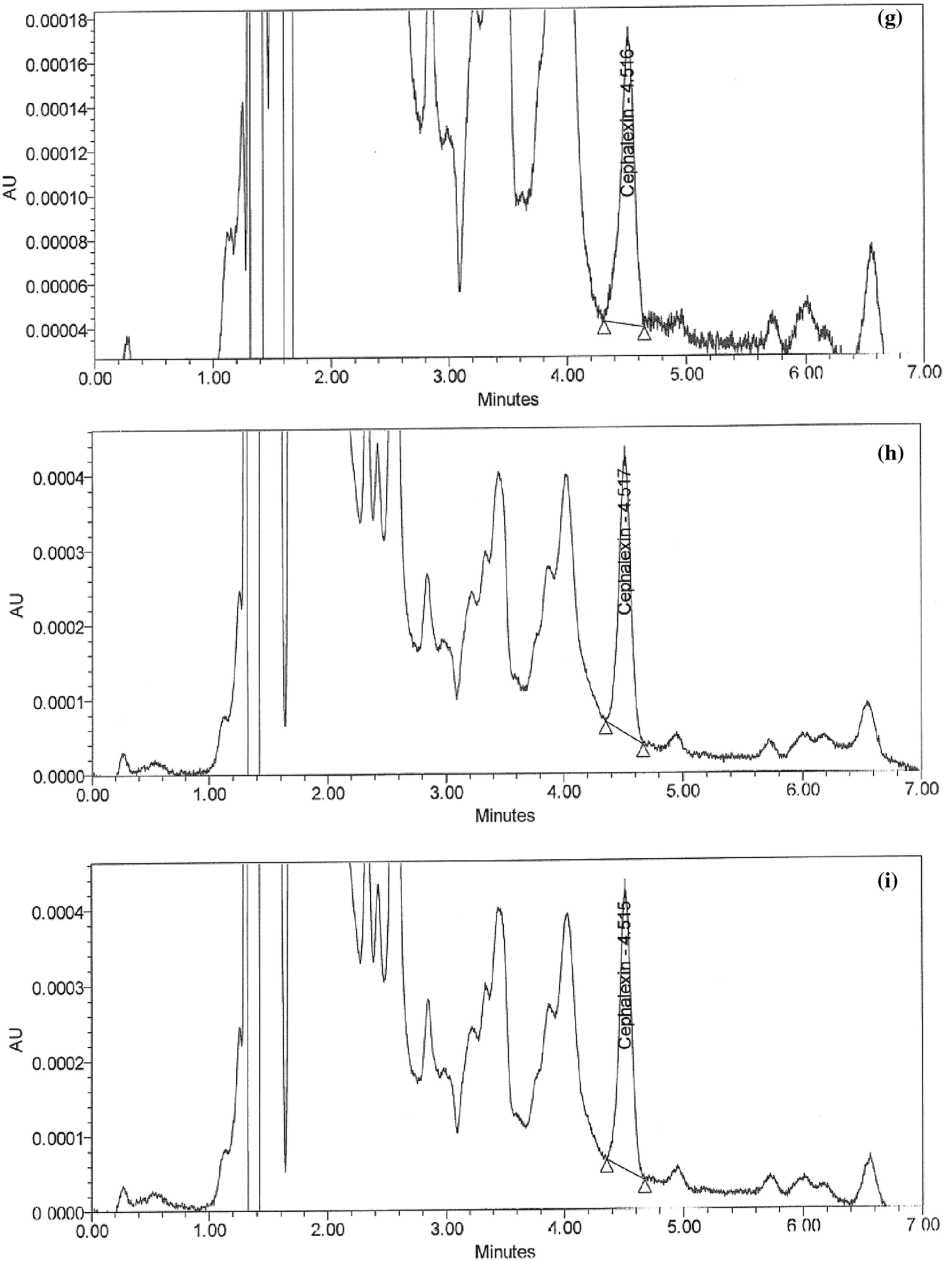

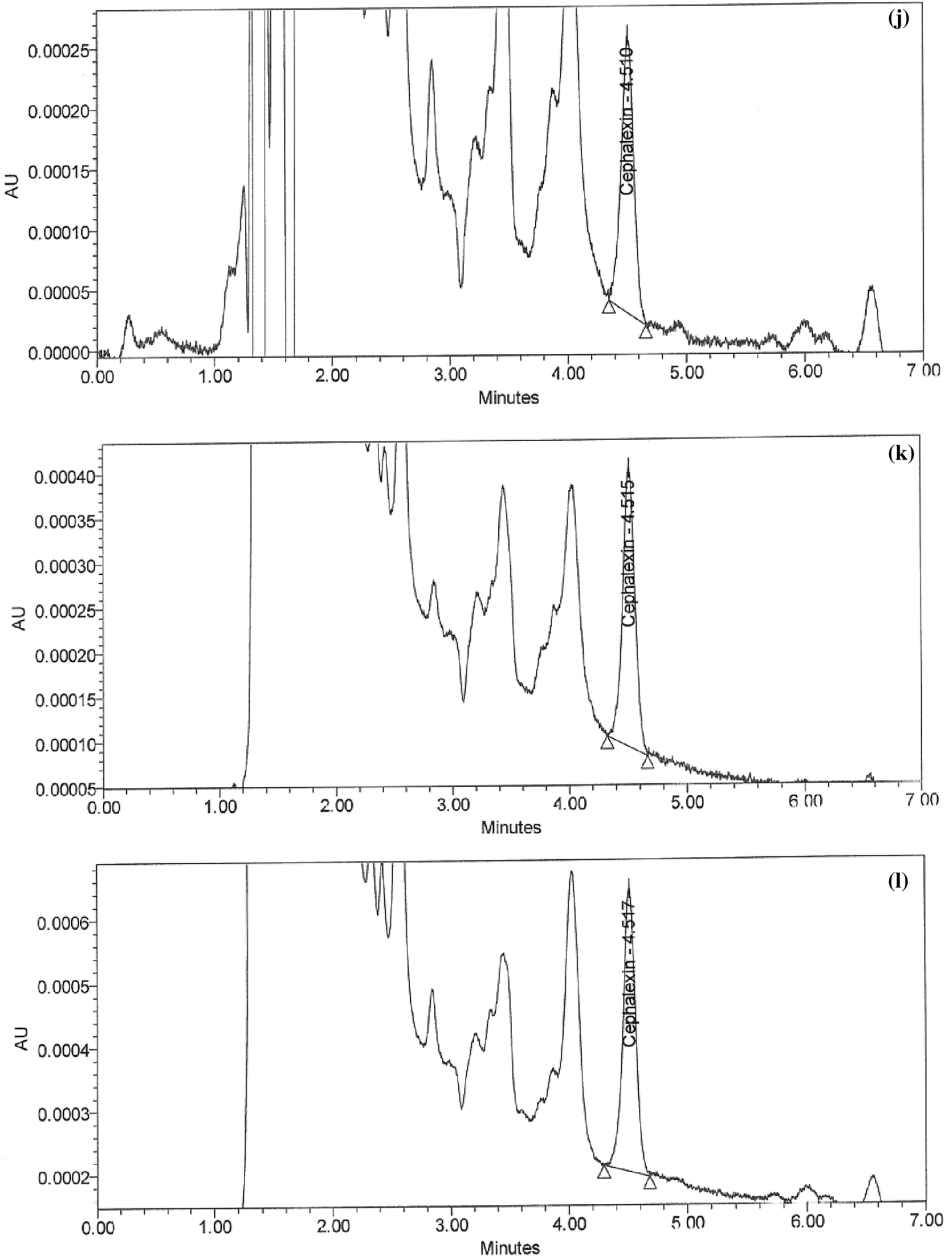
HPLC Chromatograms of (**a**) standard solution of CPH, CPH residues in (**b–g**) parts of dryer machine, and (**h**–**l**) parts of kneader machine.

Following three consecutive cycles for dirty holding time and thorough full cleaning of the capsule, suspension, and tablet lines, samples were collected at the designated sites using swab and rinse procedures and analyzed using the suggested HPLC method (Figs. 5a–i and 6a–i). Positive results were found for chemical residues of less than 5.0 ppm for the active components of CPH and CFX. Supplementary Tables S1 and S2 online show that the microbiological test findings for the samples were satisfactory, with counts of fewer than 50 cfu/25 cm^2^ and 100 cfu/ml for the swab and rinse procedures, respectively.

### Cleaning process capability

**Figure 7:**
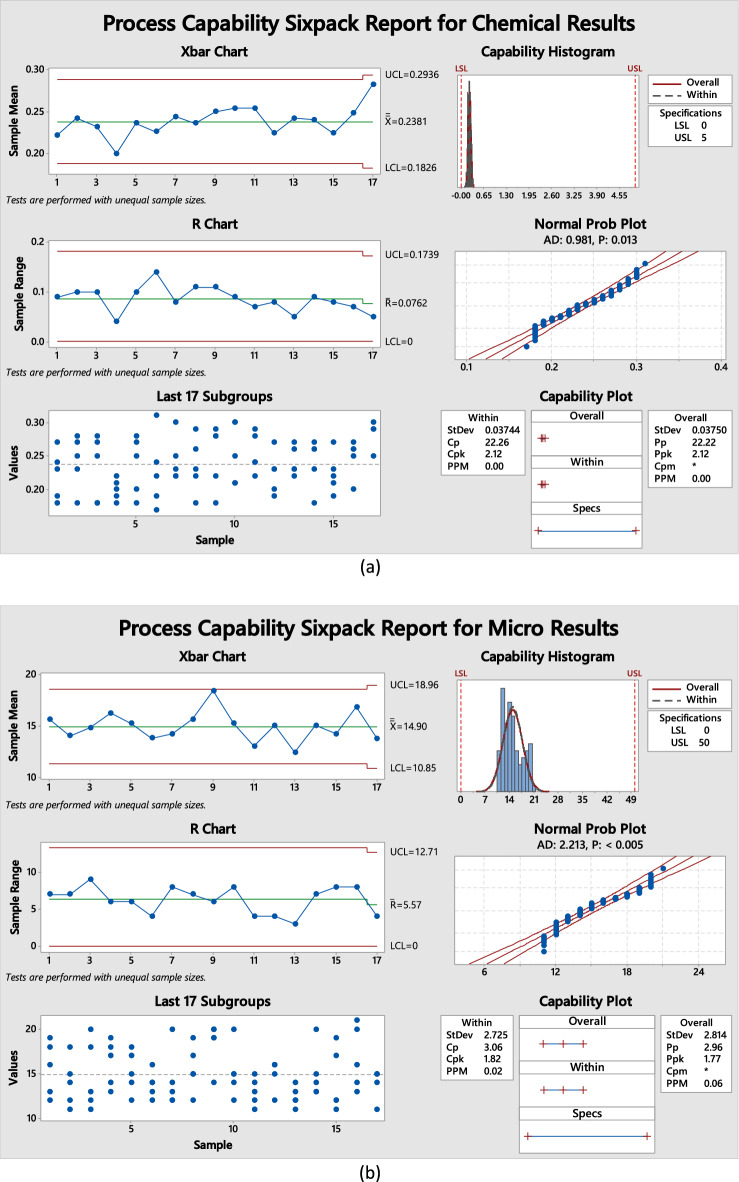
Process capability sixpack for (**a**) chemical and (**b**) micro results using Minitab18**.**

The cleaning procedure should remain valid. Supporting this with data on cleaning verification results and calculated process capabilities is recommended. Statistical trending may be performed, for instance, on the outcomes of the cleaning verification samples analyzed. The cleaning process's capability is then calculated using an appropriate statistical method. The cleaning validation samples from the capsule, suspension, and tablet lines were tested for chemicals and microbiological, and the data was processed with Minitab 18. X bar and R charts show no out-of-control points, as shown in Fig. 7a,b of the quality tools' interpreted process capability six-pack report. The data are distributed consistently and randomly from the processing center in all directions, as shown by the final 20 subgroups graphic. The procedure is generally centered on the target, and the outcomes are within the prescribed limits, as evidenced by the histogram, average probability, and capability plots. Chemical and microbiological results with a Cpk value of more than 1.33 show the significance of the suggested technique. Table 4 exhibits that the proposed method's variance, standard error, and deviation values are appropriate. The lean Six Sigma methodology has delivered outstanding results, with a process capability index score of 2.12 and 1.82 for the chemical and micro tests. This unequivocally confirms the effectiveness and suitability of the approach.

### Greenness assessment of methods

#### AMVI tool

The AMVI approach is widely recognized as a dependable and meticulous technique for evaluating liquid chromatography. It ensures that all solvents utilized, and waste generated during the analytical process are accurately measured, which is vital for obtaining precise and reliable results. To establish a standardized AMVI, it is crucial to use a specific HPLC analysis method, as it plays a critical role in achieving accurate outcomes. Moreover, it is imperative to consider and include all solvents and chemicals used in sample preparation and chromatography analysis to assess total solvent consumption accurately. Fortunately, an equation is provided that simplifies the calculations and guarantees a precise estimate of the comprehensive solvent utilization in the analytical technique.

$$\begin{aligned} {\text{The total quantity of solvent used}} & = {\text{(the sum of the solvent used for sample preparation}} \\ & \quad + {\text{the solvent used for HPLC)}}{.} \\ \end{aligned}$$

Calculating the AMVI value involves dividing the cumulative solvent usage in a specified method by the overall number of peaks of interest. Notably, a lower AMVI value can signify an increase in sustainability. This equation represents a scholarly and reliable methodology for evaluating the ecological impact of analytical methods^56^. The lower AMVI value of 128 indicates that the proposed method is more environmentally friendly, which is clear from the results shown in Table 5.

**Table 5:** Assessment of the proposed method by the AMVI tool.

Criteria	Proposed method
Solvent consumption HPLC (mL) =	**216**
Flow rate	2.0
Run time	9
Number of injections for 1 full analysis	12
Number of analytes (including impurities)	2
Solvent consumption sample prep (mL) =	**40**
Standard Prep volume (mL)	10
Number of Std. preps	1
Sample Preps volume (mL)	10
Number of Sample preps	2
System Suit volume (mL)	10
Number of System suit preps	1
Total method solvent consumption =	**256**
analytical method volume intensity	**128**
% Consumption HPLC	**84**
% Consumption preparations	**16**

Significance values are given in Bold.

#### ESA tool

To accurately assess the sustainability of an approach, it is essential to utilize the highly effective Eco-Scale tool. This tool considers various factors, such as the number of reagents used, potential hazards, energy consumption, and waste generation, and then applies a penalty score. The scores are then aggregated to determine the overall sustainability level of the approach, with a hypothetical maximum score of 100. By deducting penalty points, the overall score is reduced. An Eco-Scale score exceeding 75 is classified as "excellent green," while a score between 50 and 75 is considered "acceptable green." A score below 50 is labeled "insufficient green^57^." In our case, the system was evaluated using this tool and received an impressive eco-score of 88, indicating a high level of ecological sustainability. For a comprehensive breakdown of the penalty points, see Table 6.

**Table 6:** Penalty points for calculating the ESA score for the proposed method.

Analytical eco-scale	Penalty points
Reagents	
Purified water	0
Phosphoric acid	2
Acetonitrile	4
Instruments	
Energy for UPLC ≤ 1.5 KWh/sample	1
Occupational hazard	0
Ultrasonic	1
Vortex	1
Waste	3
Total penalty points	**12**
Eco-Scale total score	**88**

#### AGREE tool

**Figure 8:**
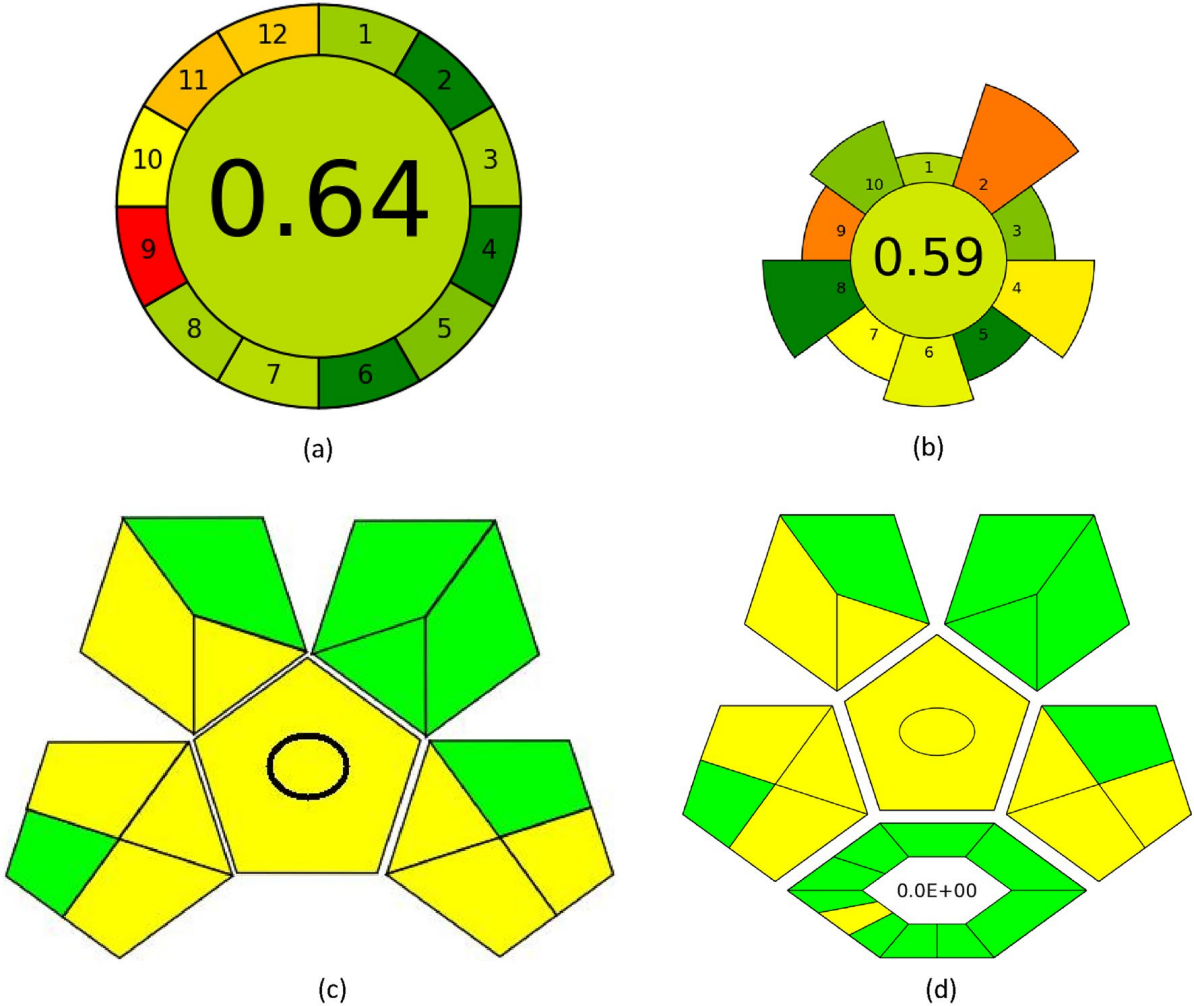
Pictograms for assessment of the "greenness" for the suggested method include (**a**) AGREE, (**b**) AGREEprep, (**c**) GAPI, and (**d**) ComplexGAPI.

The AGREE tool's evaluation strategy is genuinely all-encompassing, considering a wide range of sustainability aspects, from general factors to environmental ones. One of the critical components of the AGREE methodology is the Green Analytical Chemistry (GAC) scoring system, which is based on twelve fundamental principles. Each code is assigned a numerical score that reflects its ability to advance ecological sustainability, with values ranging from 0 to 1. The graph produced by the study uses red, yellow, and green colors to show the levels of achievement for each standard, and the size of each region in the graph is proportional to the corresponding metric that was measured. The AGREE methodology is widely used to assess environmental sustainability and is based on the 12 principles of GAC^58^, which are described in detail in Fig. S5a. The AGREE pictogram is an effective way to illustrate the concept, as shown in Fig. 8a. The central score of 0.64 is prominently displayed, along with various green hues that vary in intensity. These variations in color intensity indicate the degree of ecological sustainability achieved, making it easy to see how well an entity is doing regarding environmental responsibility.

#### AGREEprep tool

Achieving robustness in the analytical field heavily depends on the sample preparation process. The suggested method employs the AGREEprep measure, a novel approach that evaluates the ecological impact of different sample preparation techniques. By integrating this assessment procedure with the ten fundamental principles of environmentally responsible sample preparation, we are confident that our methodology demonstrates sustainability and environmental friendliness. The AGREEprep metric comprises ten discrete steps that assess individual proficiency, with a score range of 0 to 1, where a score of 1 indicates optimal performance^59^. Figure S5b displays distinctive graphic signs for each of the ten sectors. The results depicted in Fig. 8b reveal a numerical value of 0.59, indicating that our methodology is ecologically efficient.

#### GAPI tool

The environmental sustainability evaluation tool is a crucial resource for assessing proposed techniques. The comprehensive process covers all stages, from sample preparation to final analysis. It classifies the ecological impact into three levels: green, yellow, and red, further categorized as high, moderate, or low. We confidently utilize this tool to evaluate the environmental consequences associated with our analytical techniques, enabling informed decisions regarding adopting sustainable methodologies. The GAPI methodology is a unique approach used to assess the ecological sustainability of analytical procedures. It encompasses the entire analytical process, including sample preparation and final determination. It is illustrated in Fig. S6a, highlighting its fifteen descriptive components and five fundamental characteristics that distinguish it from others. The GAPI system considers all relevant factors throughout the analytical process, leading to significant efficacy when evaluating different analytical methods^60^. The results of our inquiry, as illustrated in Fig. 8c, confirm the effectiveness of this method concerning its ecological consequences.

#### ComplexGAPI tool

The ComplexGAPI metric introduces a new approach to analyzing chemical procedures. By adding an extra hexagonal layer for pre-analysis processes, this enhancement complements the existing GAPI metric, as shown in Fig. S6b. It covers various elements, including contextual circumstances, substances, solvents, sustainability, instrumentation, post-reaction processing, and purification. To evaluate the sustainability implications of each component, the GAPI system uses a color-coded scheme of red, yellow, and green, with red signifying high environmental concern, yellow indicating the moderate concern, and green representing low concern^61^. The strategies encompass various aspects, as illustrated in Fig. 8d. With its GAC attributes, the ComplexGAPI metric is an innovative and comprehensive approach to assessing methodologies.

### Method validation

Validation of the MCR and HPLC techniques was carried out as per ICH guidance^62^.

#### Linearity and range

The linearity of the HPLC and MCR techniques was determined over a range of concentrations from 0.05 to 10 and 5 to 30 ppm for CPH and CFX, respectively. There were three independent measurements of each concentration. Table 7 shows that the acquired data appeared linear, with a correlation coefficient of 0.9998 or higher.

**Table 7:** Regression and validation parameters of the proposed HPLC and MCR methods for detection of CPH and CFX.

Parameter	HPLC		MCR	
	CPH	CFX	CPH	CFX
Wavelength	254 nm	254 nm	261 nm	298 nm
Range (ppm)	0.05–10	0.05–10	5–30	5–30
Slope	50,616.6241	79,741.4019	31.7626	25.9435
Intercept	764.62	780.92	0.8037	0.2236
Correlation coefficient	0.9998	0.9998	0.9999	0.9999
Repeatability	0.1	0.2	0.07	0.05
LOD^a^ (ppm)	0.003	0.004	0.26	0.23
LOQ^a^ (ppm)	0.008	0.013	0.79	0.68

^a^Limit of detection (3.3 × σ /Slope) and a limit of quantitation (10 × σ/Slope).

#### Detection and quantitation limit

After analyzing a suitable number of blank samples and calculating the standard deviation of these responses, the LOD and LOQ were determined based on the Standard Deviation of the Blank. This process ensured accurate calculations and reliable results using the formulas (3.3σ/S) and (10σ/S), where σ refers to the standard deviation of the blank and S to the slope of the calibration curve. As shown in Table 7, the sensitivity of the proposed approaches increases as the LOQ and LOD values decrease.

#### Swab and surface recovery

To assess the swab challenge, a known concentration of CPH and CFX drugs is added to the swab, and the percentage of drugs recovered is then calculated. The range of allowable concentrations is LOQ-150%. Indirect partitions, stainless steel, plexiglass, Teflon, glass, rubber, and silicon, comparable to the equipment surface, can be spiked with the expected API. The active ingredient (API) can then be recovered and tested using the proposed HPLC technique. The swab and surface recovery results in Table 8 and Supplementary Table S3 online are acceptable, falling within the limit NLT 85% and 40%, respectively. Furthermore, the relative standard deviation (RSD) between the two concentration samples should be NMT 10%.

**Table 8:** Swab recovery results for CPH and CFX by the proposed HPLC method.

Concentration (%)	CPH				CFX			
	Plastic swab 1 (%)	Plastic swab 2 (%)	Mean (%)	RSD %	Plastic swab 1 (%)	Plastic swab 2 (%)	Mean (%)	RSD %
50	98.78	98.96	98.87	0.13	99.93	99.83	99.88	0.07
100	99.81	99.29	99.55	0.37	98.81	98.42	98.62	0.28
150	99.84	100.17	100.01	0.24	98.61	98.74	98.68	0.09
Mean recovery ± RSD			99.48	0.58			99.06	0.72

#### Precision

System Precision. The system's repeatability must be evaluated using a replicated measurement standard solution at the target concentration (5 ppm). As shown in Table 7, the system is precise since the RSD for six replicates should approach NMT 10%.

Intermediate precision (Ruggedness). Variations in the laboratory, different days, analysts, and different instruments make ruggedness readily apparent. Table S4 shows a variety of positive outcomes.

#### Robustness

The analytical suggested methods' robustness was tested to ensure that they maintained their accuracy and precision even after being subjected to deliberate changes in factors like those listed in Table S4 below, which include pH, column, temperature, wavelength, and flow rate.

#### Stability of swab holing time

The swab sample will be analyzed after being soaked in a solution for a specified period and compared to the new results. The obtained results meet the acceptance criteria for the RSD between the fresh and hold-on samples should be NMT 10%, as depicted in Supplementary Table S5 online.

#### Specificity

The clarity of assessing the analyte in the presence of potential interference components is called "specificity." It's possible that swabs or cleaning solutions are to cause for this. As shown in (Supplementary Fig. S7a,b online), blank swabs and diluent do not cause any interference.

#### System suitability

The best settings for the chromatographic conditions were chosen using BBD optimization. The theoretical plate count, resolution, tailing factor, and retention time probes from the system suitability test evaluated the HPLC technique. See Table S6 for the data that supports the HPLC technique.

## Conclusion

The QbD strategy was used to develop and validate low-cost, new, specific, and Eco-friendly RP-HPLC and MCR techniques for detecting pharmaceutical drug residues of CPH and CFX in manufacturing equipment. Using the fewest possible experimental runs, BBD and RSM were employed to enhance chromatographic parameters for maximum resolution and minimum retention time for both drugs. Protecting human health and the environment, cutting unnecessary waste, and saving money on solvents and labor for analysts are just a few advantages of using green solvents. Further, the MCR approach was applied to the CPH and CFX binary mixtures to resolve interference without requiring derivative processes or complex software. These methods are valid and appropriate for quality control without expensive HPLC equipment. Six Sigma was also implemented to eliminate manufacturing costs, enhance product quality and reliability, and keep the process relatively close to the target mean. The proposed method has been successfully validated according to the ICH criteria. It may be easily implemented in any environment requiring analysis of the examined drugs in pure or dosage form and detection of active pharmaceutical drug residues in manufacturing equipment.

### Supplementary Information

Supplementary Information.

## Data Availability

All information gathered and analyzed during this endeavor is included in this article.
