# Supplementary material for: Condition optimization of eco-friendly RP-HPLC and MCR methods via Box–Behnken design and six sigma approach for detecting antibiotic residues
Source: Sci Rep. 2023 Sep 21;13:15729. doi: 10.1038/s41598-023-40010-1 (PMC10514345; doi:10.1038/s41598-023-40010-1)
Supplement: Supplementary file 1 — Supplementary Information. [file 41598_2023_40010_MOESM1_ESM.docx]

**Condition Optimization of Eco-friendly RP-HPLC and MCR Methods via Box-Behnken Design and Six Sigma Approach for Detecting Antibiotic Residues**

**Tahani Y. A. Alanazi^1^,** **Rami Adel Pashameah^2^, Ammena Y. Binsaleh^3^, Mahmoud A. Mohamed ^4^, Hoda A. Ahmed^5^, and Hossam F. Nassar ^6*^**

^1^Chemistry Department, Faculty of Science, University of Ha’il, P.O. Box 2440, Ha’il 81451, Saudi Arabia.

^2^ Department of Chemistry, Faculty of Applied Science, Umm Al-Qura University, Makkah al-Mukarramah, Saudi Arabia.

^3^ Department of Pharmacy Practice, College of Pharmacy, Princess Nourah bint Abdulrahman University, P.O. Box 84428, Riyadh 11671, Saudi Arabia.

^4^Hikma Pharmaceutical Company, Beni-Suef, Egypt.

^5^ Department of Chemistry, Faculty of Science, Cairo University, Cairo 12613, Egypt.

^6*^Department of Environmental Sciences and Industrial Development, Faculty of Post Graduate Studies for Advanced Sciences, Beni-Suef University, Beni‑Suef, Egypt.

^1^E-mail : [thay44@hotmail.co.uk](mailto:thay44@hotmail.co.uk) ; ^2^E-mail : [rapasha@uqu.edu.sa](mailto:rapasha@uqu.edu.sa) ; ^3^E-mail : [aysaleh@pnu.edu.sa](mailto:aysaleh@pnu.edu.sa) ;^4^E-mail : [ch.mahmoud88@gmail.com](mailto:ch.mahmoud88@gmail.com); ^5^E-mail : [ahoda@sci.cu.edu.eg](mailto:ahoda@sci.cu.edu.eg) ; ^6^*E-mail : [hossamnassarnrc@gmail.com](mailto:hossamnassarnrc@gmail.com).

**Abstract**

A precise, Eco-friendly, and highly sensitive RP-HPLC method was employed using quality-by-design principles to concurrently identify cephalexin and cefixime residues in the manufacturing machines using a hypersil BDS C18 column (250 × 4.6 mm, 5μm) at wavelength 254 nm. The Box-Behnken design was applied to obtain the best chromatographic conditions with the fewest possible trials. Three independent factors viz organic composition, flow rate, and pH were used to assess their effects on the responses' resolution and retention time. Overlay plot and desirability functions were implemented to predict responses of the high resolution and relatively short retention time using a mobile phase composed of acidic water: acetonitrile (85:15, v/v) at pH 4.5 adjusted by phosphoric acid with a flow rate of 2.0 mL/min. The spectral overlapping of the drugs was successfully resolved by the mean centering ratio (MCR) spectra approach at 261nm and 298 nm for cephalexin and cefixime, respectively. Good linearity results were obtained for the suggested HPLC and MCR methods over the concentration range of (0.05–10 ppm) and (5-30 ppm) with a detection limit of 0.06,0.06, 0.26, and 0.23 ppm~~,~~ and quantitation limits of 0.17, 0.18, 0.79, and 0.68 ppm for cephalexin and cefixime, respectively, with a correlation coefficient of ≥ 0.9998 and good swab recovery results of 99-99.5%. A process capability index was accomplished for chemical and micro results, illustrating that both are extremely capable. The suggested method was effectively validated using ICH recommendations.

| \| \| 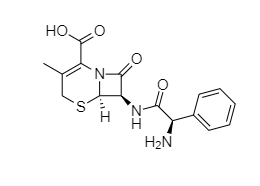 \| \| --- \| \| \| --- \| --- \| \| **(a)** Cephalexin **(CPH)**  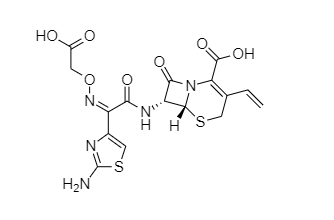 \| \| Cefixime **(CFX)(b)**  **Figure S1**. Chemical structure of (a) CPH and (b) CFX**.** \| |
| --- | --- | --- | --- | --- |

(a)


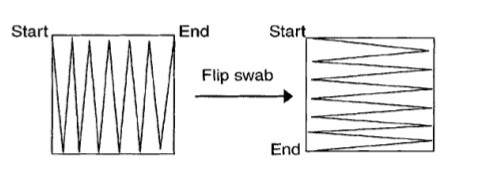


(b)

**Figure S2**. Swab sampling technique of (a) Strokes horizontally and vertically and (b) Zigzag manner**.**

**Figure S3**. Zero-order absorption spectra of 10 µg/mL of CFX, 10 µg/mL of CPH, and 10 µg/mL of Mixture using solvent as blank.


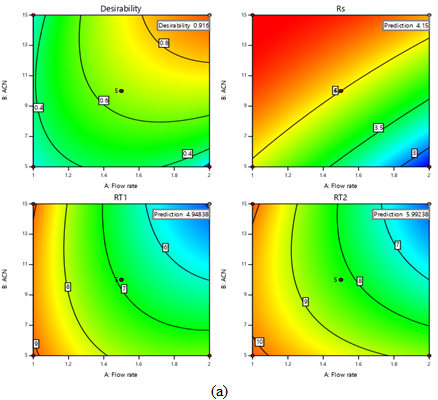


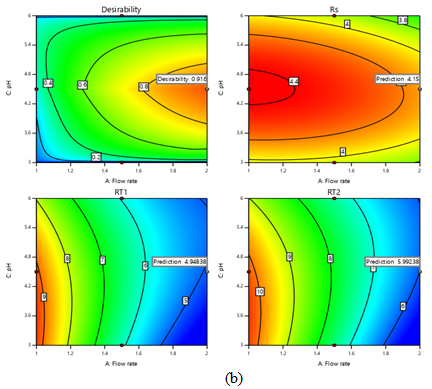


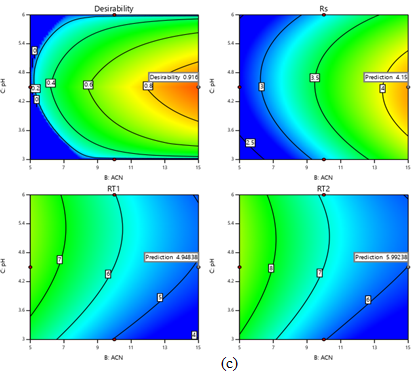


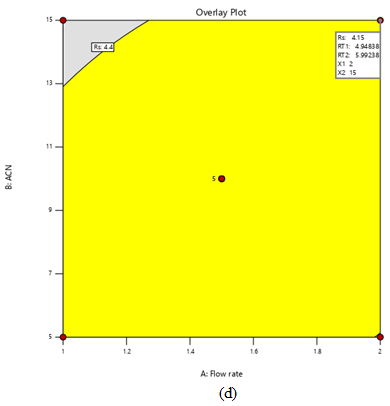


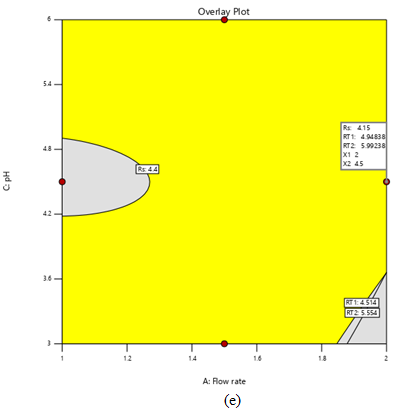


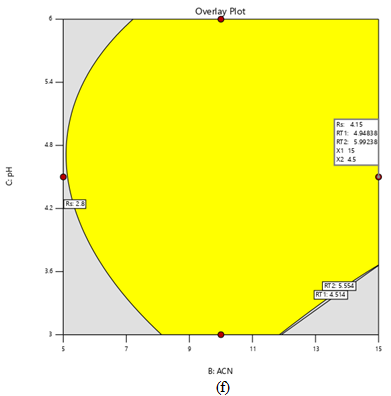


**Figure S4.** Desirability (a-c) and overlay plots predicted responses (d-f).


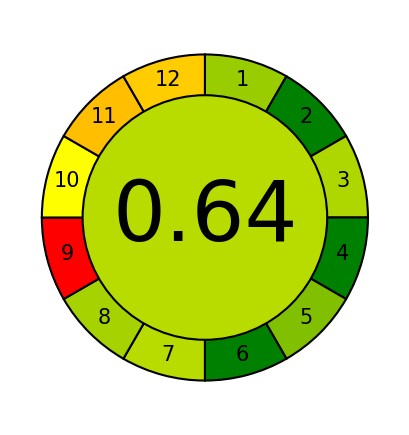
**Analytical Greenness report sheet**

24/06/2023 22:55:09

**Criteria**

**Score**

**Weight**

1

. Direct analytical techniques should be applied to avoid sample

treatment.

0.7

2

2

. Minimal sample size and minimal number of samples are goals.

1.0

2

. If possible, measurements should be performed in situ.

3

0.66

2

. Integration of analytical processes and operations saves energy and

4

reduces the use of reagents.

1.0

2

. Automated and miniaturized methods should be selected.

5

0.75

2

. Derivatization should be avoided.

6

1.0

2

7

. Generation of a large volume of analytical waste should be avoided, and

proper management of analytical waste should be provided.

0.64

2

. Multi-analyte or multi-parameter methods are preferred versus methods

8

using one analyte at a time.

0.68

2

9

. The use of energy should be minimized.

0.0

2

. Reagents obtained from renewable sources should be preferred.

10

0.5

2

11

. Toxic reagents should be eliminated or replaced.

0.37

2

. Operator's safety should be increased.

12

0.4

2

(a)


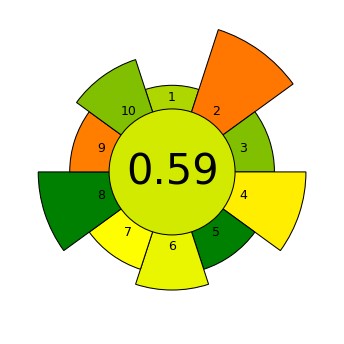
**AGREEprep**

Analytical Greenness Metric for Sample Preparation

24/06/2023 23:55:52

**# Criterion Score Weight**

| 1. | **Sample preparation placement** | 0.66 | 1 |
| --- | --- | --- | --- |
|  | Sample preparation placement: On-line/In situ |  |  |
| 2. | **Hazardous materials** | 0.23 | 5 |
|  | Mass [g] or volume [mL] of problematic materials: 2 |  |  |
| 3. | **Sustainability, renewability, and reusability of materials** | 0.75 | 2 |
|  | > 75% of reagents and materials are sustainable or renewable |  |  |
| 4. | **Waste** | 0.47 | 4 |
|  | Mass [g] or volume [mL] of waste: 2.7 |  |  |
| 5. | **Size economy of the sample** | 1.0 | 2 |
|  | Mass [g] or volume [mL] of the sample: 0.1 |  |  |
| 6. | **Sample throughput** | 0.54 | 3 |
|  | Hourly sample throughput: 10 |  |  |
| 7. | **Integration and automation** | 0.5 | 2 |
|  | No. of sample prep. steps: 2 steps or fewer; degree if automation: Semi-automated systems |  |  |
| 8. | **Energy consumption** | 1.0 | 4 |
|  | Approximate energy consumption per analysis [W]: 1.5 |  |  |
| 9. | **Post-sample preparation configuration for analysis** | 0.25 | 2 |
|  | Liquid chromatography, gas chromatography with quadrupole detection, etc. |  |  |
| 10. | **Operator's safety** | 0.75 | 3 |
|  | No. of distinct hazards: 1 hazard |  |  |

(b)

**Figure S5.** Evaluation report for (a) AGREE and (b) AGREEprep for the suggested method.


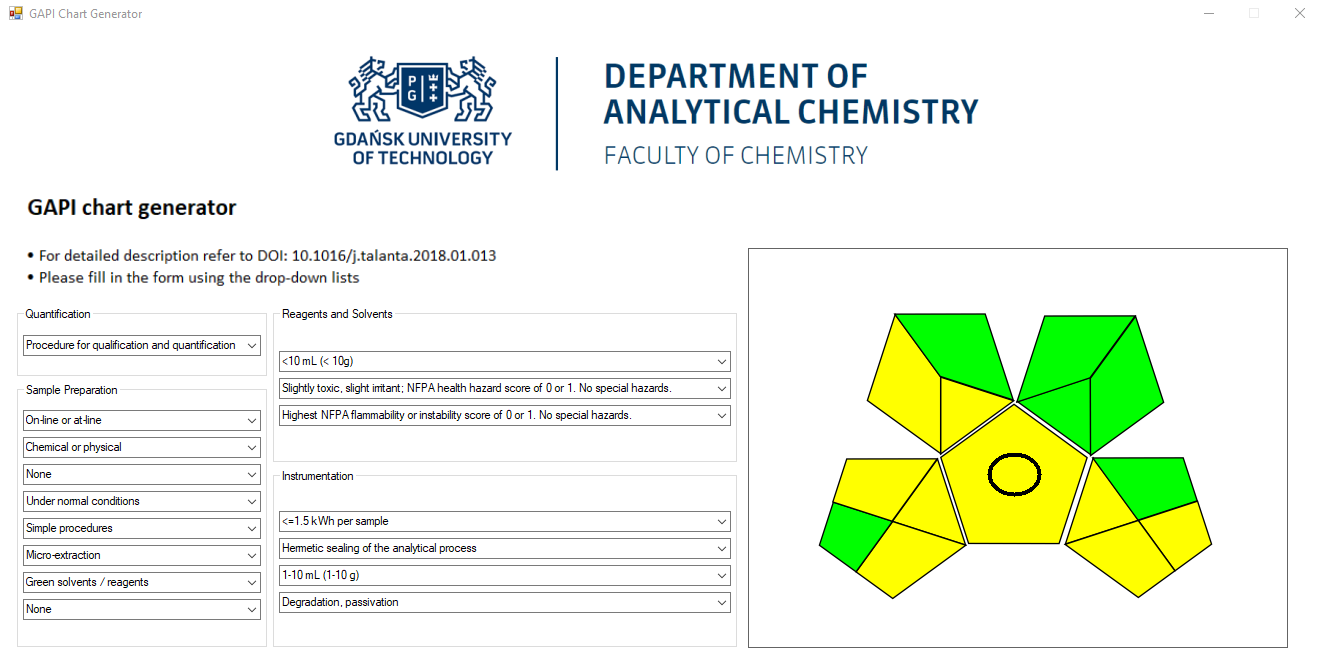


(a)


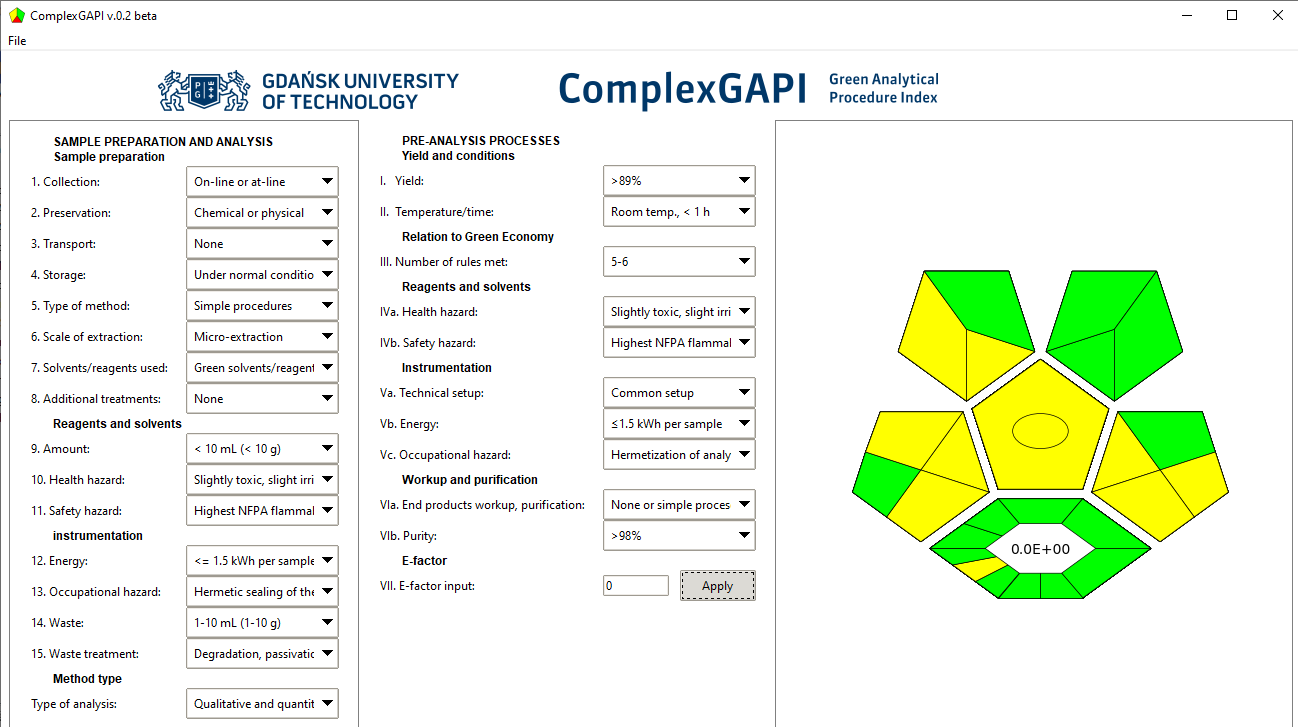


(b)

**Figure S6.** Assessment report for (a) GAPI and (b) ComplexGAPI for the proposed method.


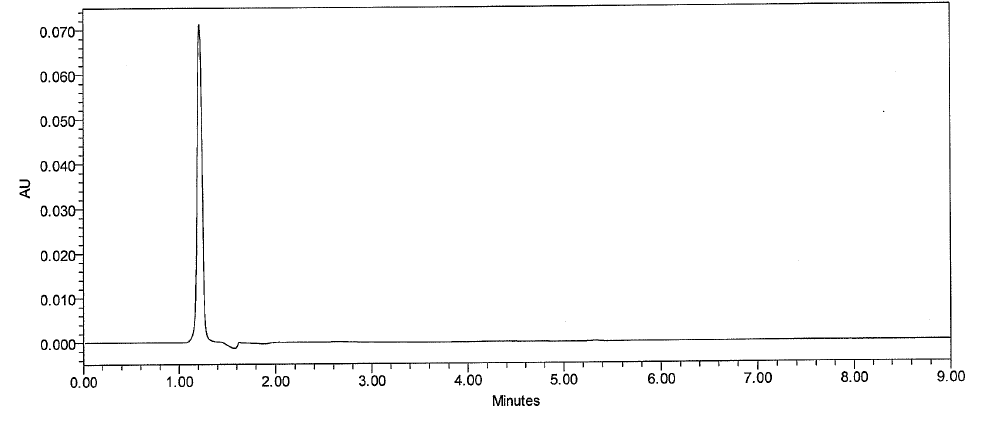


(a)


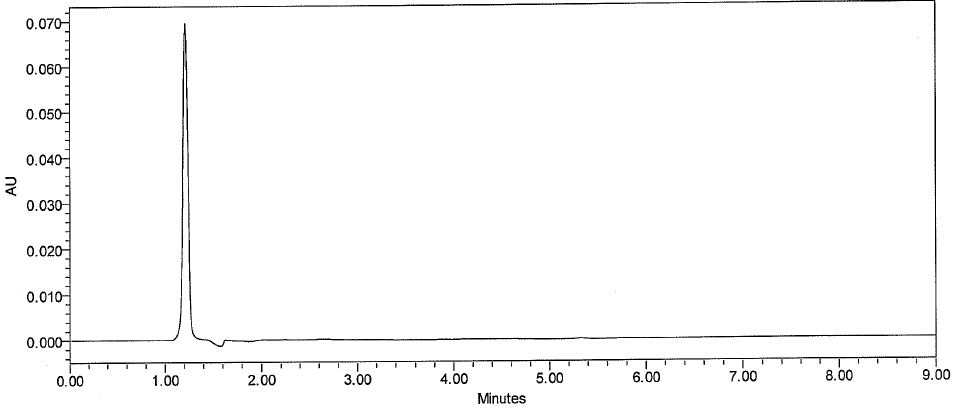


(b)

**Figure S7**. HPLC Chromatogram of (a) diluent (b) plastic swab**.**

**Table S1.** Results of dirty holding time in capsule, suspension, and tablet lines.

| **Cycle#** | **Cycle # 1** | **Cycle # 2** | **Cycle # 3** | **Limit** |
| --- | --- | --- | --- | --- |
| **Fluid bed dryer** |  | | | **NMT 50 (cfu/25cm^2^)** |
| Number of hours before clean | 64 hr | 88 hr | 87 hr |  |
| Machine parts | Micro results (cfu/25cm^2^) | | |  |
| Product Pan left | 11 | 3 | 4 |  |
| Product Pan right | 7 | 9 | 2 |  |
| Product Pan bottom | 9 | 4 | 3 |  |
| Filter Housing | 12 | 1 | 1 |  |
| Dryer body left | 8 | 1 | 4 |  |
| Dryer body right | 9 | 1 | 3 |  |
| Filter ring | 4 | 1 | 1 |  |
| **High-Speed Mixer (Kneader)** |  | | |  |
| Number of hours before clean | 67 hr | 90 hr | 90 hr |  |
| Machine parts | Micro results (cfu/25cm^2^) | | |  |
| Cover of Kneader | 10 | 1 | 1 |  |
| Inner Surface | 9 | 1 | 1 |  |
| Impeller | 7 | 1 | 3 |  |
| Chopper | 8 | 1 | 1 |  |
| Discharge Gate | 9 | 1 | 1 |  |
| **Sifter** |  | | |  |
| Number of hours before clean | 62 hr | 66 hr | 70 hr |  |
| Machine parts | Micro results (cfu/25cm^2^) | | |  |
| Sieve | 5 | 3 | 1 |  |
| Pan | 10 | 1 | 2 |  |
| Product Discharge | 8 | 1 | 1 |  |
| **Compression** |  | | |  |
| Number of hours before clean | 38 hr | 67 hr | 166 hr |  |
| Machine parts | 1 | 1 | 2 |  |
| Material Hopper | 5 | 1 | 4 |  |
| Machine Body | 1 | 1 | 8 |  |
| Feeder | 20 | 1 | 1 |  |
| Tablet Discharge | 28 | 1 | 1 |  |
| Dies | 1 | 5 | 1 |  |
| Punches | 1 | 1 | 1 |  |
| Tray | 1 | 3 | 1 |  |
| Product Container | 1 | 1 | 6 |  |
| Product Container Feeder | 1 | 1 | 3 |  |
| Metal Detector Out | 2 | 1 | 1 |  |
| Tablet Scrapper | 1 | 1 | 1 |  |
| Metal Detector in | 1 | 1 | 1 |  |
| Inner Side of Window Cover I | 1 | 1 | 1 |  |

**Table S2.** Results of full clean in capsule, suspension, and tablet lines.

| **Cycle#** | **Cycle # 1** | | | **Cycle # 2** | | | **Cycle # 3** | | |
| --- | --- | --- | --- | --- | --- | --- | --- | --- | --- |
| **Fluid bed dryer** |  | | | | | | | | |
| Batches before cleaning # | 1 | | | 1 | | | 2 | | |
| Batch# | 2104 | | | 2105 | | | 2107 | | |
| Number of Days# | 1 | | | 1 | | | 2 | | |
| Visual Cleaning Check | Clean without any traces of previous product | | | | | | | | |
| Machine parts | API | Micro | Detergent | API | Micro | Detergent | API | Micro | Detergent |
| Product Pan left | 0.19 ppm | 19 cfu/25cm^2^ | N/A | 0.18 ppm | 18 cfu/25cm^2^ | N/A | 0.18 ppm | 11 cfu/25cm^2^ | N/A |
| Product Pan right | 0.23 ppm | 18 cfu/25cm^2^ |  | 0.28 ppm | 12 cfu/25cm^2^ |  | 0.18 ppm | 17 cfu/25cm^2^ |  |
| Product Pan bottom | 0.27 ppm | 16 cfu/25cm^2^ |  | 0.27 ppm | 11 cfu/25cm^2^ |  | 0.20 ppm | 14 cfu/25cm^2^ |  |
| Filter Housing | 0.24 ppm | 13 cfu/25cm^2^ |  | 0.25 ppm | 20 cfu/25cm^2^ |  | 0.19 ppm | 18 cfu/25cm^2^ |  |
| Dryer body left | 0.18 ppm | 12 cfu/25cm^2^ |  | 0.18 ppm | 12 cfu/25cm^2^ |  | 0.22 ppm | 19 cfu/25cm^2^ |  |
| Dryer body right | 0.25 ppm | 15 cfu/25cm^2^ |  | 0.28 ppm | 18 cfu/25cm^2^ |  | 0.21 ppm | 13 cfu/25cm^2^ |  |
| Filter ring | 0.23 ppm | 14 cfu/25cm^2^ |  | 0.27 ppm | 13 cfu/25cm^2^ |  | 0.18 ppm | 18 cfu/25cm^2^ |  |
| Rinse | N/A | 8 cfu/ml | 0.825µs/cm | N/A | 2 cfu/ml | 0.817µs/cm | N/A | 1 cfu/ml | 0.825µs/cm |
| **Kneader** |  | | | | | | | | |
| Machine parts | API | Micro | Detergent | API | Micro | Detergent | API | Micro | Detergent |
| Cover of Kneader | 0.25 ppm | 15 cfu/25cm^2^ | N/A | 0.24 ppm | 12 cfu/25cm^2^ | N/A | 0.22 ppm | 12 cfu/25cm^2^ | N/A |
| Inner Surface | 0.27 ppm | 14 cfu/25cm^2^ |  | 0.19 ppm | 14 cfu/25cm^2^ |  | 0.23 ppm | 14 cfu/25cm^2^ |  |
| Impeller | 0.20 ppm | 17 cfu/25cm^2^ |  | 0.22 ppm | 16 cfu/25cm^2^ |  | 0.25 ppm | 12 cfu/25cm^2^ |  |
| Chopper | 0.28 ppm | 12 cfu/25cm^2^ |  | 0.31 ppm | 14 cfu/25cm^2^ |  | 0.22 ppm | 20 cfu/25cm^2^ |  |
| Discharge Gate | 0.17 ppm | 13 cfu/25cm^2^ |  | 0.30 ppm | 13 cfu/25cm^2^ |  | 0.18 ppm | 17 cfu/25cm^2^ |  |
| Rinse | N/A | 12 cfu/ml | 0.808µs/cm | N/A | 28 cfu/ml | 1.040µs/cm | N/A | 7 cfu/ml | 0.838µs/cm |
| **Sifter** |  | | | | | | | | |
| Machine parts | API | Micro | Detergent | API | Micro | Detergent | API | Micro | Detergent |
| Sieve | 0.23 ppm | 15 cfu/25cm^2^ | N/A | 0.26 ppm | 19 cfu/25cm^2^ | N/A | 0.28 ppm | 19 cfu/25cm^2^ | N/A |
| Pan | 0.29 ppm | 12 cfu/25cm^2^ |  | 0.28 ppm | 19 cfu/25cm^2^ |  | 0.18 ppm | 20 cfu/25cm^2^ |  |
| Product Discharge | 0.22 ppm | 15 cfu/25cm^2^ |  | 0.22 ppm | 20 cfu/25cm^2^ |  | 0.29 ppm | 14 cfu/25cm^2^ |  |
| Rinse | N/A | 12 cfu/ml | 0.689µs/cm | N/A | 1 cfu/ml | 0.856µs/cm | N/A | 12 cfu/ml | 1.033µs/cm |
| **Compression** |  | | | | | | | | |
| Machine parts | API | Micro | Detergent | API | Micro | Detergent | API | Micro | Detergent |
| Material Hopper | 0.30 ppm | 14 cfu/25cm^2^ | N/A | 0.20 ppm | 12 cfu/25cm^2^ | N/A | 0.21 ppm | 17 cfu/25cm^2^ | N/A |
| Machine Body | 0.21 ppm | 15 cfu/25cm^2^ |  | 0.23 ppm | 16 cfu/25cm^2^ |  | 0.23 ppm | 19 cfu/25cm^2^ |  |
| Feeder | 0.30 ppm | 12 cfu/25cm^2^ |  | 0.23 ppm | 14 cfu/25cm^2^ |  | 0.22 ppm | 12 cfu/25cm^2^ |  |
| Tablet Discharge | 0.25 ppm | 15 cfu/25cm^2^ |  | 0.22 ppm | 12 cfu/25cm^2^ |  | 0.19 ppm | 11 cfu/25cm^2^ |  |
| Dies | 0.21 ppm | 20 cfu/25cm^2^ |  | 0.23 ppm | 12 cfu/25cm^2^ |  | 0.26 ppm | 16 cfu/25cm^2^ |  |
| Punches | 0.24 ppm | 13 cfu/25cm^2^ |  | 0.27 ppm | 11 cfu/25cm^2^ |  | 0.27 ppm | 20 cfu/25cm^2^ |  |
| Tray | 0.24 ppm | 11 cfu/25cm^2^ |  | 0.26 ppm | 13 cfu/25cm^2^ |  | 0.25 ppm | 21 cfu/25cm^2^ |  |
| Product Container | 0.28 ppm | 12 cfu/25cm^2^ |  | 0.23 ppm | 14 cfu/25cm^2^ |  | 0.26 ppm | 14 cfu/25cm^2^ |  |
| Product Container Feeder | 0.29 ppm | 15 cfu/25cm^2^ |  | 0.27 ppm | 13 cfu/25cm^2^ |  | 0.2 ppm | 13 cfu/25cm^2^ |  |
| Metal Detector Out | 0.22 ppm | 14 cfu/25cm^2^ |  | 0.26 ppm | 13 cfu/25cm^2^ |  | 0.29 ppm | 11 cfu/25cm^2^ |  |
| Tablet Scrapper | 0.27 ppm | 15 cfu/25cm^2^ |  | 0.26 ppm | 20 cfu/25cm^2^ |  | 0.25 ppm | 15 cfu/25cm^2^ |  |
| Metal Detector in | 0.19 ppm | 16 cfu/25cm^2^ |  | 0.18 ppm | 15 cfu/25cm^2^ |  | 0.3 ppm | 14 cfu/25cm^2^ |  |
| Inner Side of Window | 0.23 ppm | 16 cfu/25cm^2^ |  | 0.27 ppm | 12 cfu/25cm^2^ |  | 0.29 ppm | 15 cfu/25cm^2^ |  |
| Rinse | N/A | 1 cfu/ml | 0.737µs/cm | N/A | 1 cfu/ml | 0.870µs/cm | N/A | 1 cfu/ml | 0.905µs/cm |

**Table S3.** Surface recovery results for CPH and CFX by the proposed HPLC method.

|  | **CPH** | | | | **CFX** | | | |
| --- | --- | --- | --- | --- | --- | --- | --- | --- |
| **Surface** | **Plastic swab 1** | **Plastic swab 2** | **Mean** | **RSD %** | **Plastic swab 1** | **Plastic swab 2** | **Mean** | **RSD %** |
| Stainless Surface | 84.30 % | 84.44 % | 84.37 % | 0.12 | 91.61 % | 91.27 % | 91.44 % | 0.26 |
| Plexi-Glass Surface | 94.99 % | 94.83 % | 94.91 % | 0.12 | 99.68 % | 98.44 % | 99.06 % | 0.89 |
| Teflon Surface | 88.72 % | 88.51 % | 88.62 % | 0.17 | 96.39 % | 96.41 % | 96.4 % | 0.01 |
| Glass Surface | 88.66 % | 88.67 % | 88.67 % | 0.01 | 91.63 % | 91.66 % | 91.65 % | 0.02 |
| Rubber Surface | 97.08 % | 97.41 % | 97.24 % | 0.24 | 96.46 % | 96.45 % | 96.45 % | 0.01 |
| Silicon Surface | 88.42 % | 88.62 % | 88.52 % | 0.16 | 88.34 % | 88.50% | 88.42 % | 0.13 |
| Indirect Partition | 97.21 % | 97.19 % | 97.20 % | 0.01 | 98.56 % | 98.54% | 98.55 % | 0.01 |

**Table S4.** Ruggedness and robustness for drugs by the proposed methods.

| **Parameter** | **HPLC** | | **MCR** | | **Limit %** |
| --- | --- | --- | --- | --- | --- |
|  |  |  |  |  |  |
|  | **CPH** | **CFX** | **CPH** | **CFX** | **RSD ≤ 2.0%** |
| Day to Day | 1.34 | 1.47 | 1.12 | 1.19 |  |
| Analyst to Analyst | 1.15 | 1.49 | 0.91 | 0.83 |  |
| Column to Column | 1.63 | 1.78 | **-** | **-** |  |
| Flow rate change (±0.1 mL/min) | 1.59 | 1.67 | **-** | **-** |  |
| pH changes of mobile phase (±0.2) | 1.23 | 1.51 | **-** | **-** |  |
| Wavelength change (±2.0) nm | 1.57 | 1.34 | 1.25 | 1.38 |  |
| Column temperature change (±2.0) ^ₒ^C | 1.45 | 1.38 | **-** | **-** |  |

**Table S5.** Results for the stability of swab holding time for CPH and CFX by the proposed HPLC method.

|  | **CPH** | | **CFX** | |
| --- | --- | --- | --- | --- |
| **Holding time** | **Plastic swab recovery** | **RSD %** | **Plastic swab recovery** | **RSD %** |
| Freshly prepared | 99.25 % | - | 97.03 % | - |
| After 24 hours | 100.93 % | 1.19 % | 95.94 % | 0.80 % |
| After 48 hours | 90.03 % | 6.89 % | 95.08 % | 1.44 % |
| After 72 hours | 93.76 % | 4.02 % | 89.79 % | 5.48 % |

**Table S6.** System compatibility testing of the proposed HPLC technique.

| **Reference values** | **HPLC** | | **Item** |
| --- | --- | --- | --- |
|  | **CFX** | **CPH** |  |
| T ≤ 2 | 1.12 | 1.20 | Tailing factor |
| RSD ≤1% | 0.26 | 0.33 | Injection precision |
| N > 2000 | 6248 | 8425 | Number of theoretical plates (N) |
| Rs ≥ 1.5 | 4.4 | - | Resolution (Rs) |
